# Increasing knowledge of HIV status in a country with high HIV testing coverage: Results from the Botswana Combination Prevention Project

**DOI:** 10.1371/journal.pone.0225076

**Published:** 2019-11-25

**Authors:** Mary Grace Alwano, Pamela Bachanas, Lisa Block, Michelle Roland, Baraedi Sento, Stephanie Behel, Refeletswe Lebelonyane, Kathleen Wirth, Faith Ussery, William Bapati, Catherine Motswere-Chirwa, William Abrams, Gene Ussery, James A. Miller, Ebi Bile, Peter Fonjungo, Agisanag Kgwadu, Molly Pretorius Holme, Lisetta Del Castillo, Tendani Gaolathe, Kelebemang Leme, Nokuthula Majingo, Shahin Lockman, Joseph Makhema, Naomi Bock, Janet Moore

**Affiliations:** 1 U.S. Centers for Disease Control and Prevention, Division of Global HIV/AIDS, Gaborone, Botswana; 2 U.S. Centers for Disease Control and Prevention, Division of Global HIV/AIDS, Atlanta, Georgia, United States of America; 3 Northrop Grumman, Atlanta, Georgia, United States of America; 4 Tebelopele HIV Counseling and Testing Center, Gaborone, Botswana; 5 Botswana Ministry of Health and Wellness, Gaborone, Botswana; 6 Harvard T.H. Chan School of Public Health, Boston, Massachusetts, United States of America; 7 Botswana Harvard AIDS Institute Partnership, Gaborone, Botswana; 8 Brigham and Women’s Hospital, Boston, Massachusetts, United States of America; Sefako Makgatho Health Sciences University, SOUTH AFRICA

## Abstract

**Introduction:**

Achieving widespread knowledge of HIV-positive status is a crucial step to reaching universal ART coverage, population level viral suppression, and ultimately epidemic control. We implemented a multi-modality HIV testing approach to identify 90% or greater of HIV-positive persons in the Botswana Combination Prevention Project (BCPP) intervention communities.

**Methods:**

BCPP is a cluster-randomized trial designed to evaluate the impact of combination prevention interventions on HIV incidence in 30 communities in Botswana. Community case finding and HIV testing that included home and targeted mobile testing were implemented in the 15 intervention communities. We described processes for identifying HIV-positive persons, uptake of HIV testing by age, gender and venue, characteristics of persons newly diagnosed through BCPP, and coverage of knowledge of status reached at the end of study.

**Results:**

Of the 61,655 eligible adults assessed in home or mobile settings, 13,328 HIV-positive individuals, or 93% of the estimated 14,270 positive people in the communities were identified through BCPP. Knowledge of status increased by 25% over the course of the study with the greatest increases seen among men (37%) as compared to women (19%) and among youth aged 16–24 (77%) as compared to older age groups (21%). Although more men were tested through mobile than through home-based testing, higher rates of newly diagnosed HIV-positive men were found through home than mobile testing.

**Conclusions:**

Even when HIV testing coverage is high, additional gains can be made using a multi-modality HIV testing strategy to reach different sub-populations who are being missed by non-targeted program activities. Men and youth can be reached and will engage in community testing when services are brought to places they access routinely.

## Introduction

The HIV epidemic remains a major public health problem globally, with 1.8 million new infections occurring in 2017 [1]. Sub-Saharan Africa is the most affected region, experiencing 65% of these new HIV infections [1]. Although significant progress has been made in treating HIV and preventing new infections over the past three decades, major challenges have been encountered in reaching the global goal of epidemic control. In 2014, UNAIDS launched the ambitious 90-90-90 targets in an attempt to curb the tide of new infections. These goals were: by the year 2020, that 90% of people living with HIV (PLHIV) would know their status; 90% of PLHIV who knew their status would be sustained on antiretroviral therapy (ART); and 90% of those on ART would be virally suppressed.

Several studies have concluded that achieving widespread coverage of appropriately targeted HIV testing so that 90% of PLHIV know their status is one of the main obstacles to achieving universal ART coverage, population-level viral suppression, and ultimately epidemic control [2, 3–5]. UNAIDS estimates that 2.7 million HIV-positive people in Eastern and Southern Africa remain to be identified to achieve the 1^st^ 90 [6]. Studies conducted in sub-Saharan Africa have highlighted barriers to achievement of the first UNAIDS target [2–8]. Social factors such as stigma, fear of discrimination, concerns about disclosure and perceived low risk have been reported as obstacles for HIV testing among youth, key populations and men. Economic factors including lack of transport, distance to health facilities, inability to miss work, mobility, migration, and health system issues (e.g. shortage of supplies and staff, long waiting queues) remain major barriers for many people to access HIV testing [2, 7–8].

Community-based HIV testing interventions address some of the barriers to HIV testing and significantly increase testing uptake and coverage [2, 7–19]. The HPTN 071 (PopART) study in Zambia reported HIV testing uptake of 72.2%, increasing knowledge of HIV status from ~50% to ~90% after one year of community-based door-to-door HIV Testing and Counseling (HTC) [10]. Similarly, a systematic review and meta-analysis of community and facility-based HIV testing found that community-based HTC modalities achieved higher coverage than facility-based testing overall, with home-based testing (70%) and community campaigns (76%) having the highest population coverage, while facility voluntary counseling and testing (15%) and provider-initiated testing and counseling (18%) attained the lowest coverage [7]. In addition to removing barriers that occur with facility-based testing (e.g. long waiting time, transport costs, fear of disclosure, and stigma), community-based testing modalities also reached more first-time testers, HIV-infected persons with high CD4 counts, and more males and youth than facility-based HTC [7,8]. However, community-based testing can be more costly than integrated facility-based testing depending upon the country, setting and intensity of the intervention [7,8].

Botswana, a middle- income country with a population of approximately 2 million, has the second highest HIV prevalence in the world (estimated at 22% of adults) [6]. With several decades of HIV testing available to its citizens, Botswana has made significant progress towards reaching the 1^st^ 90. Early adoption of opt-out HIV testing and implementation of a national Antiretroviral Treatment (ART) program have resulted in relatively high testing rates and knowledge of HIV-positive status [12]. HIV incidence, however, has remained above 1% in the country [20]. Effective strategies for reaching people who do not know their HIV status in a country like Botswana where both population HIV testing coverage and HIV incidence are high have not been identified.

The Botswana Combination Prevention Project (BCPP) implemented and evaluated intensive HIV community-based testing strategies in addition to linkage to care activities and expanded ART provision, with the goal of reducing HIV incidence. The objectives of this paper are to describe the process by which HIV-positive persons were identified in the 15 BCPP intervention communities, the uptake of HIV testing by age, sex and venue, and characteristics of newly-identified HIV-positive persons. We compare home-based and mobile testing modalities to determine which is more successful at identifying HIV-positive persons by age and sex. In addition, increases in knowledge of HIV-positive status from study start to study end using community-based HIV testing modalities are reported.

## Methods

### Design

The BCPP was a cluster-randomized HIV prevention trial that evaluated the impact of a combination of prevention interventions on population-level HIV incidence in Botswana. A full description of the study design is available elsewhere [12]. The trial was conducted between October 2013 and December 2017 in 30 rural or peri-urban communities, which had an average population size of approximately 6,000. Fifteen communities were assigned to the intervention arm and 15 to the standard of care arm. Community was defined for purposes of this study as having a health center that provided HIV care and treatment, HIV testing and PMTCT; clear geographical boundaries that allowed determination of whether a household was in or outside of the study community; and of sufficient size to meet sample size criteria. One of the most important goals of the BCPP study was for 90% or more of PLHIV in the intervention communities to know their HIV status by the end of the study. Using a multi-modality HIV testing strategy composed of community-based home and mobile testing, we attempted to cover 100% of the intervention communities with HIV testing services. Data from the intervention communities only are presented here.

### Participants

All persons 16–64 years old who were community residents (spent at least 3 nights/month in the community) were assessed through an intake and asked if they were HIV-positive. Those who knew their HIV-positive status were asked for documentation of status and treatment regimen, if on ART. Persons who did not know their status, who did not have documentation of an HIV-positive status, or who did not have documentation of a negative HIV test within the preceding 3 months were offered HIV testing. All HIV testing and data collection were conducted by trained local lay counselors who were fluent in Setswana, English and other local languages. Although HIV testing was offered to all residents regardless of citizenship, only data from citizens or spouses of citizens were included in this report.

### Modality of HIV testing

HIV testing services were offered in the homes and through mobile services that targeted venues where different segments of the population congregate in order to reach the remaining HIV-positive population not found through the Botswana national testing program.

#### Home-based HIV testing

Community mobilizers went door-to-door and throughout the community alerting residents to the availability of HIV testing and other services. Counselors received lists of all household plots for each of the intervention communities (obtained from Google maps and Botswana 2011 census data). GPS coordinates were used to locate the households on handheld tablets. Given that many Botswana residents are highly mobile and often move between multiple residences including urban/peri-urban homes close to jobs, homes in their local village/birthplace, cattle posts and farmlands, counselors labeled households as inhabited/regularly occupied, inhabited but no one found at home after 3 visits, or seasonally/sporadically occupied. Counselors returned to households up to three times over a two-month period in order to find residents at home for assessment of HIV status and HIV testing if indicated. The study teams conducted evening and weekend HIV testing and made appointments for home-based testing for times when absent residents would be home.

Household residents age 16 years or older were asked to participate in the BCPP intervention. Those who agreed completed a brief intake questionnaire which consisted of questions on demographics, prior HIV testing history and HIV test results. Persons who did not know they were HIV-positive or had no documentation of test results were offered HIV testing services. Verbal consent for HIV testing (the standard in Botswana) was also obtained and documented on the intake form. The Botswana national testing algorithm was followed using point-of-care finger stick testing with parallel Unigold (Trinity Biotech, Ireland) and KHB (Shanghai Kehua Bio-engineering Co Ltd, China) rapid tests.

#### Mobile testing

In the first round of HIV testing (which lasted approximately two years), home and mobile testing were offered concurrently using an intermittent campaign approach. Mobile testing was made available throughout communities at locations where the general population was frequently found including markets, transport hubs, and tents set up close to home-based testing for individuals who preferred the privacy of testing outside of the home. After completion of the Round 1 testing campaign, review of the data showed that men and young people were underrepresented among participants reached with this strategy. Therefore, during Round 2, a more targeted outreach approach to mobile HIV testing was prioritized over home-based testing and was offered on an ongoing basis by community counselors in the intervention communities for the remainder of the study. Strategies to find men and young people specifically were emphasized, including going to high traffic areas where men and young people congregate, workplaces, markets, shebeens/bars, cattle posts/farms, etc. In addition, testing was provided at community events, and multi-disease health fairs were conducted in collaboration with the health facilities and community traditional leadership.

Persons accessing mobile venues completed the same study procedures as persons participating in the home. Participants were asked to complete an intake questionnaire and were interviewed to determine if they already knew their HIV positive status. If they did not know their status or if they knew their status but had no documentation, they were offered an HIV test.

All newly-identified or known HIV-infected persons not on ART identified in home or mobile venues were counseled on the benefits and importance of early care and treatment for their health and the health of their partners and children, as well as their eligibility for treatment. They were also given referral forms and appointment dates at their local Ministry of Health (MOH) clinics.

### Lab Quality Assurance (QA)

To ensure quality control for the field-based HIV rapid testing, 100% of field samples from the 1^st^ community, 100% of all HIV-positive and 5–10% of HIV- negative samples from all subsequent communities were retested for HIV in the reference laboratory with enzyme-linked immunosorbent assay (ELISA) using the Vironostika Uniform II Plus O (bioMerieux, Marcy l’Etoile, France) in the first community. Remnant microtube specimens were transported to the reference laboratory for DBS preparation, stored and used for retesting according to manufacturer’s guidelines. In subsequent communities, Bio-Rad Genetic Systems^™^ HIV-1/HIV-2 *PLUS O* EIA (Bio-Rad Laboratories, Redmond, WA, USA) was used in accordance with the Botswana national HIV testing algorithm. Western blot testing in cases of discordant results was planned; however, no discordant results were observed. In addition, starting in June 2016, all persons referred with a positive HIV test underwent verification HIV rapid testing at the clinic.

### Ethics approval

The study was approved by the Centers for Disease Control and Prevention Institutional Review Board (Protocol #6475) and the Botswana Health Research and Development Committee (Institutional Review Board of the Botswana Ministry of Health and Wellness). The study was monitored by an Independent Data and Safety Monitoring Board.

### Data collection, variable measurement, and analyses

#### Data collection

All questionnaires and results of HIV tests were collected on encrypted handheld tablets, which were synchronized daily with the research database and removed from the tablet after synchronization to maximize data confidentiality. Botswana citizens have a unique individual identification number issued by the government (Omang) which is used by the health care system. This unique identifier was used to prevent double counting of persons re-testing in different venues. It was also used to gather supporting documentation of a person’s HIV status from electronic medical records in Botswana.

1. **Measurement of Study Variables.** Algorithms were designed to measure the following outcomes: total assessed, testing yield, total HIV-positives identified, total HIV-positives at baseline, total HIV-positives identified during BCPP, an estimate of all HIV-positives, and coverage of knowledge of positive HIV status. Total persons assessed includes all unique individuals with a valid identifier who consented to an intake in a home or mobile location and whose HIV status was determined to be either HIV-negative or HIV-positive using both test results and/or documentation.
2. Testing Yield was the total number of people who newly tested HIV-positive through BCPP community testing as a percentage of the total number of people receiving an HIV test. For repeat testers, a person’s final positive or negative HIV status was used in the determination of yield. The testing venue (home or mobile) where the final status was obtained was used in the testing yield by venue analysis.
3. Total number of HIV-positive persons identified by study end included all unique persons with a valid identifier who either tested HIV-positive or had documentation of being HIV-positive in either home or mobile venues.
4. The number of HIV-positive persons before study start (baseline) was defined as all HIV-positive persons completing an intake with documentation or clinical records indicating an HIV-positive status prior to BCPP beginning testing campaigns in each community.
5. Number of HIV-positive persons identified during the course of the study included persons directly tested in BCPP through home and mobile. Also included were HIV-positive persons who received their diagnosis during the course of BCPP in their community but were not tested by BCPP testing services.
6. Estimate of the number of HIV-positive community residents was derived from a 20% random household sample in the 15 study communities. The most recent Google satellite imagery available prior to study start was used to identify plots with structures. Plots were classified as residential and habitable if the geographic area appeared to be used for living and/or sleeping by at least one person (e.g. house or hut with roof, and some evidence of being inhabited, such as curtains in windows, livestock). Based upon household enumeration in the 20% sample, the proportion of plots with structures that were residential, habitable and regularly occupied was determined. The number of regularly occupied households and the average number of household residents were extrapolated to the remaining 80% of regularly-occupied households to determine the total number of study eligible residents in each community by age and sex. The estimated study eligible population in each community was standardized to the 2011 Botswana Census by age and sex. The HIV prevalence of each community was also derived from the 20% random household sample in each community and was computed by age and sex. The community-specific age and sex distributions from the 2011 Botswana Census were used to adjust the HIV prevalence estimates obtained from the 20% random household sample. The estimated total number of HIV-positive persons by age and sex in each community was calculated by multiplying the community-specific study eligible residents and adjusted HIV prevalence by age and sex. Ninety-five percent confidence intervals were derived using a nonparametric bootstrapping procedure (n = 500 bootstrap samples) conditional on community.
7. Overall coverage of knowledge of positive HIV status was calculated by dividing the total number of HIV-positive persons identified through BCPP testing efforts by the total number of HIV-positive adults estimated to be living in the 15 intervention communities.
8. Percent increase in knowledge of HIV-positive status was calculated using the difference between the number of HIV-positive persons who knew their status at study start and the number who knew their status at study end, dividing by the persons who knew their status at study start, and multiplying by 100.

#### Analysis

SAS 9.4 was used for all statistical analyses. SAS PROC SURVEYFREQ was used to produce frequencies, adjusting for clustering of observations within communities. Bivariate tests of association were conducted using Rao-Scott Chi-square tests, with a critical alpha level of 0.05. SAS PROC SURVEYLOGISTIC was used to test for association between sociodemographic factors of interest and HIV positivity. These models produced unadjusted and adjusted odds ratios (ORs) and 95% confidence limits.

## Results

### Assessment of HIV status

**Fig 1:**
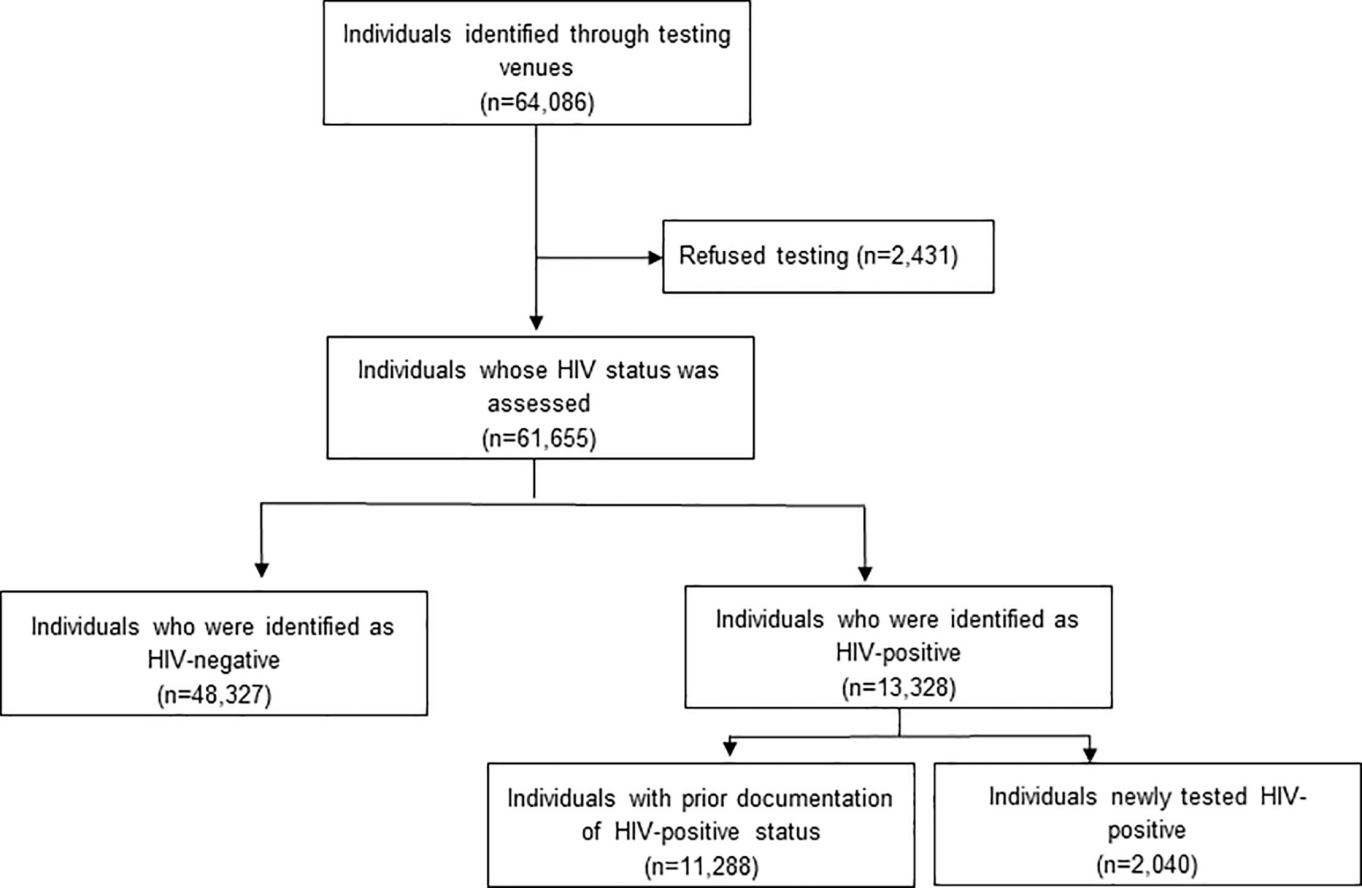
Consort diagram of the assessment of HIV status among participants identified in the Botswana Combination Prevention Project.

A total of 64,086 unique adults were interviewed in home or mobile venues. Among those interviewed, 11,288 (18%) had prior documentation of an HIV-positive status, 1,519 (2%) had documentation of a negative test result in the past 3 months, and the remaining 51,279 (80%) were offered an HIV test. Of those offered an HIV test, 2431 (5%) refused testing and the remaining 48,848 were tested (Fig 1). Of persons assessed for HIV status (61,655), 33,763 were assessed in the home and 27,892 were assessed through mobile venues (Table 1). A higher percentage of women were assessed in the home (62%) than mobile venues (38%), and a slightly higher percentage of men were assessed in mobile venues (55%) than in the home (45%). Among persons 16–24 and 25–34, a slightly higher percentage was assessed in mobile venues (54% and 51%, respectively) than in the home (46% and 49%, respectively). Persons with Primary or lower levels of education were more likely to be assessed in the home versus mobile (67% vs 33%) whereas similar numbers of persons with secondary levels of education or higher were assessed in home and mobile venues (51% vs 49%). Unemployed persons more likely to be assessed in the home (58% vs 42%), whereas 50% of employed persons were assessed in both home and mobile venues. Single persons were slightly more likely to be assessed in mobile vs home settings (52% vs 48%), and married people more likely to be assessed in the home (63% vs 37%).

**Table 1:** Demographic characteristics of persons assessed for HIV in BCPP by HIV testing venue.

	Total Assessed 61,655	Assessed in Home 33,763	Assessed in Mobile 27,892	p*
**Sex**				P<0.0001
**Female**	33,961	21,165 (62)	12,796 (38)	
**Male**	27,694	12,598 (45)	15,096 (55)	
**Age (years)**				P<0.0001
16–24	19,182	8,747 (46)	10,435 (54)	
25–34	18,124	8,807 (49)	9,317 (51)	
35–64	24,349	16,209 (67)	8,140 (33)	
**Education Level**				P<0.0001
Primary or Lower	15,123	10,191 (67)	4,932 (33)	
Secondary or Higher	46,496	23,536 (51)	22,960 (49)	
**Employment Status**				P<0.0001
Employed (Full/Part Time)	23,921	11,866 (50)	12,055 (50)	
Not Employed	37,658	21,847 (58)	15,811 (42)	
**Relationship Status**				P<0.0001
Single/Never Married	46,007	23,695 (52)	22,312 (48)	
Cohabitating/Married	13,840	8,739 (63)	5,101 (37)	
Divorced/Separated/Widowed	1,799	1,321 (73)	478 (27)	

Values are presented as frequency (%).

*p value calculated using the Rao-Scott modified Chi square test.

### HIV testing

Of the 48,848 who were HIV tested through BCPP, 51% were female and 49% were male; 37% were 16–24, 31% were 25 to 34, 32% were between 35 to 64. More persons were tested in mobile settings (54%) as compared to the home (46%). Among those testing in home or mobile venues, 2,040 (4.2%) were HIV-positive.

Table 2 shows the analysis of HIV-positive yield (i.e., number newly testing positive among all persons tested) by age, sex, and testing venue. Overall, there was a higher yield of new positives among persons aged 25–64 (5.4%) compared to persons aged 16–24 (2.1%) [AOR = 2.64 (2.15, 3.14), p<0.001]. Yield was also higher for persons testing in the home (4.9%) compared to mobile venues (3.6%) [AOR = 1.31 (14, 1.50); p = 0.001]. Sex alone was not associated with yield of testing, however, there was a sex by age interaction (F = 40.4, p<0.001), with higher yield observed among men than among women across each age stratum except among those aged 16–24 where yield was greater for women than men. There was also a sex by testing venue interaction (F = 22.4, p< 0.001), with higher yield found among men tested in the home than in mobile settings. For women, there were no significant differences in yield by venue testing across all age strata.

**Table 2:** Percentage of newly identified HIV-positive persons among all persons tested by sex, age & testing venue.

	TOTAL FEMALES	TOTAL MALES		FEMALES 16–24	FEMALES 25–34	FEMALES 35–64	MALES 16–24	MALES 25–34	MALES 35–64
**Tested in** **Home** **N = 22,613**	13,275	9,338		4,317	3,550	5,408	3,443	2,659	3,236
**HIV+ in Home** **N = 1,105** **(4.9%)**	608 (4.6%)	497 (5.3%)		122 (2.8%)	190 (5.4%)	296 (5.5%)	48 (1.4%)	157 (5.9%)	292 (9.0%)
**Tested in** **Mobile** **N = 26,235**	11,767	14,468		5,131	3,703	2,933	5,111	5,171	4,186
**HIV+ in** **Mobile** **N = 935** **(3.6%)**	455 (3.9%)	480 (3.3%)		155 (3.0%)	162 (4.4%)	138 (4.7%)	51 (1.0%)	196 (3.8%)	233 (5.6%)

### Total number of HIV-positive persons identified

Of the 61,655 persons assessed through home or mobile testing, 13,328 were found to be HIV-positive; 11,288 (85%) had documentation of their HIV-positive status at study intake (86% on ART; 14% not on ART), and 2,040 (15%) learned their status through HIV testing conducted by BCPP. All HIV-positive persons not on ART, previously diagnosed and newly tested, were referred to treatment in their community.

**Fig 2:**
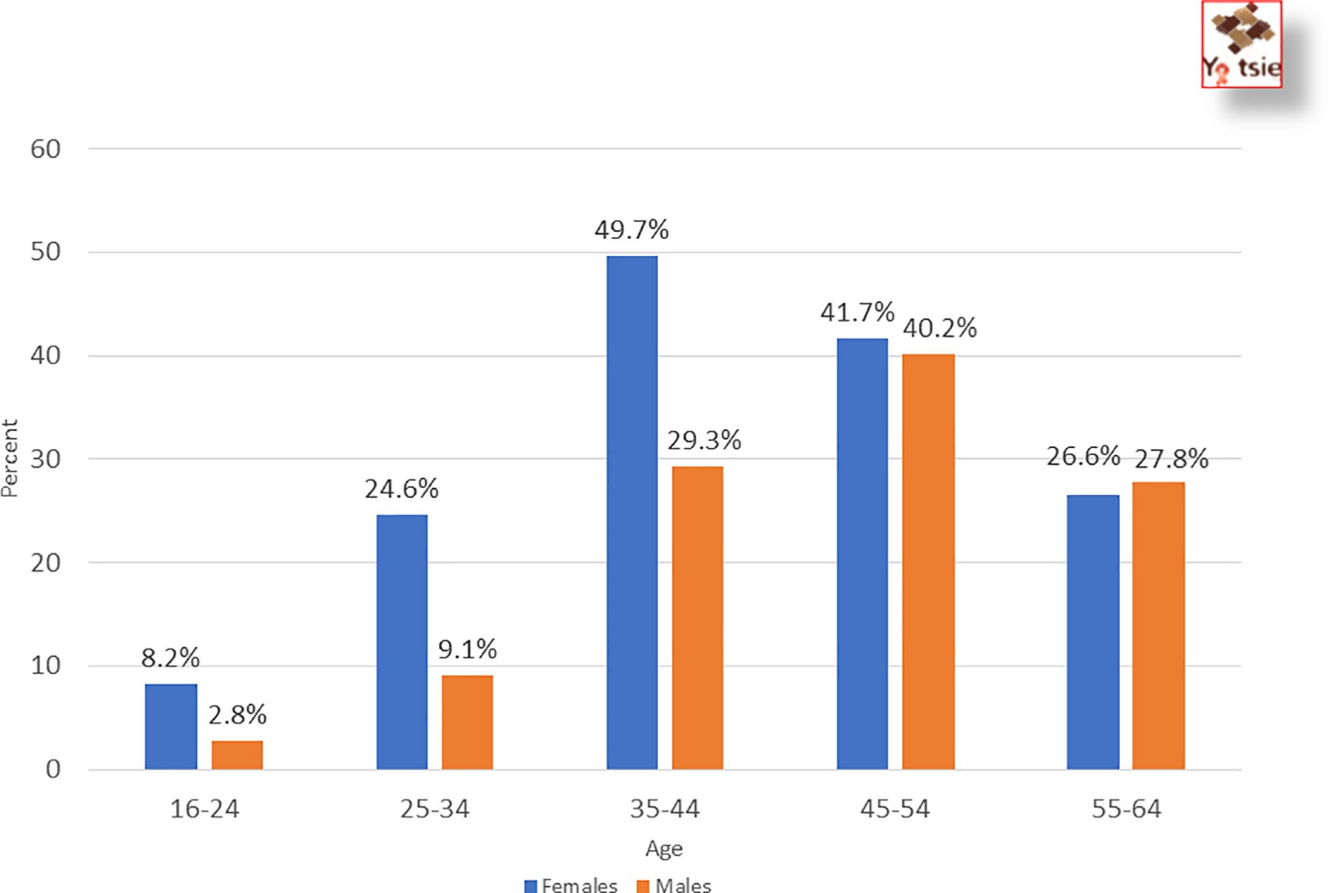
Proportion of HIV-positive persons among persons assessed for HIV by sex & age in 15 intervention communities.

Women had a higher rate of HIV-positivity (26%) than men (15.6%; p<0.001). The proportion of HIV-positive women peaked in the 35–44 year age group with nearly half of women found to be HIV-positive (49.7%) (Fig 2). Positivity among men did not peak until age 45–55 with 40% of men HIV-positive. The proportion of women found to be HIV-positive in all age categories between 16 and 44 years is 2 to 3 times higher than the proportion of HIV-positives among men in those age categories (Rao Scott chi square = 90.37, p<0.001). In the mid-forty age range, the proportion of HIV-positive persons among all women and all men are similar (Rao Scott chi square = 0.26, p = 0.61).

**Fig 3:**
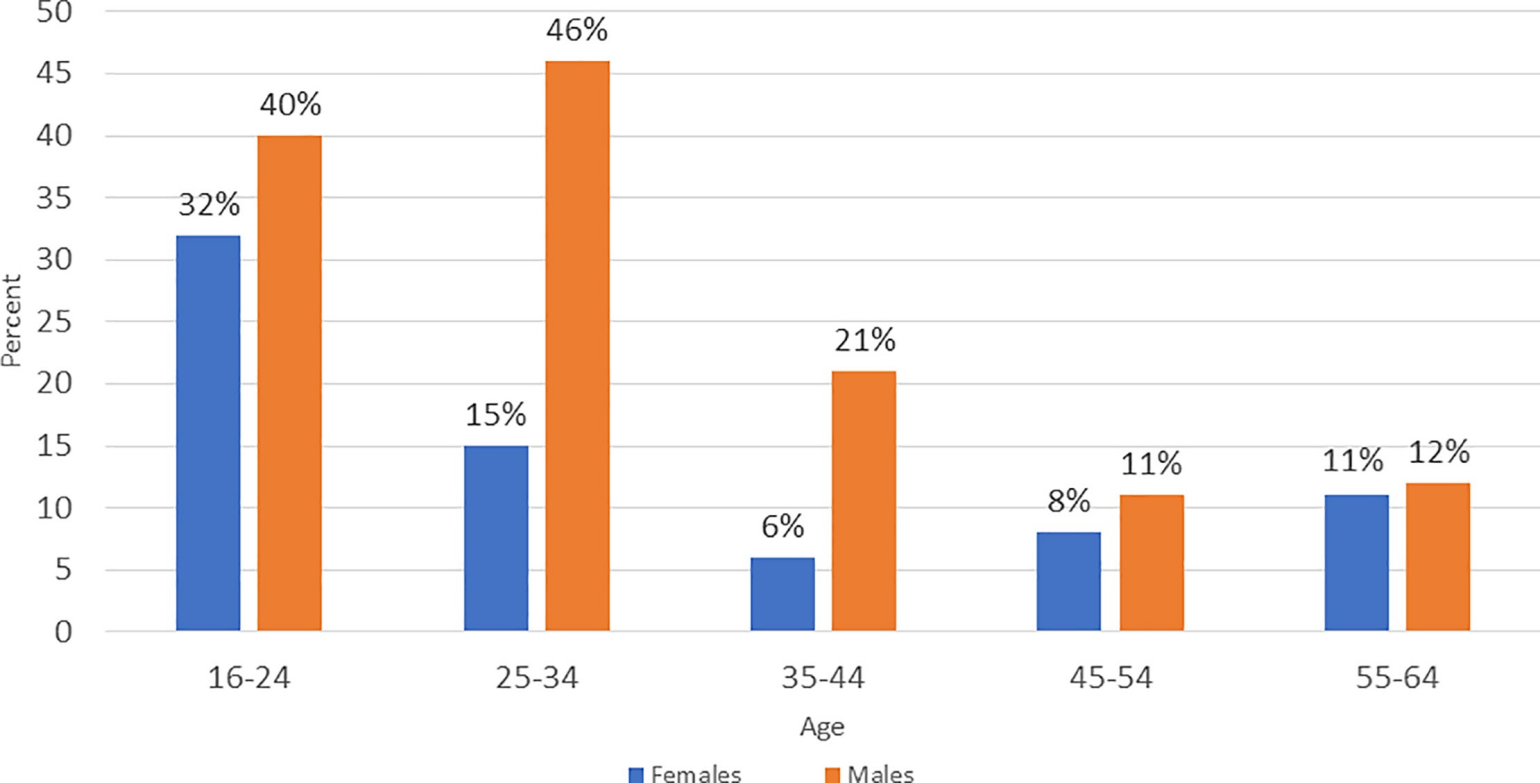
Percentage of new HIV-positive persons among all HIV-positive persons identified by sex & age in 15 intervention communities.

The percentage of newly diagnosed females was about one-third of all positive women identified in the 16–24 age range but rapidly reduced to 15% by age 25–34 and continued to decline through age 64 (Fig 3). The percent of newly diagnosed males among all positive males identified was 41% in the 16–24 age range, climbed to 46% in the 25–34 age range, and then began to drop at age 35 to 64.

### HIV-positive population estimate

The satellite maps yielded an estimated 35,066 plots with structures in the 15 intervention communities. From the household enumeration in the random sample, 78% of the plots had structures that were residential, habitable and regularly occupied. The average household size for plots containing one household was 3.8 members and 2.8–2.5 members for multi-household plots. This information was extrapolated to the remaining 80% of regularly-occupied households to determine the total number of study eligible residents in each community. Based upon the extrapolation, the 15 intervention communities had an estimated 99,147 total residents, and 54% (54,569) of household members were estimated to meet age and residency criteria for the study.

HIV case finding and testing of household members in the 20% random sample found an overall HIV prevalence of 29%. Because older and female community residents were over-represented in the 20% sample, HIV prevalence was standardized for each community based on the proportion in each age and sex category found in the 2011 Botswana Census. The estimated total number of HIV-positive persons by age and sex in each community and adjusted HIV prevalence by age and sex produced 14,270 estimated HIV-infected residents in the intervention communities (95% CI [13,497,^15^,041]).

### Population-level knowledge of HIV-positive status

Before the BCPP testing intervention was implemented, BCPP identified 10,703 HIV-positive persons who already knew their HIV status, or 75% of the estimated 14,270 positive persons in the 15 communities. By study end, BCPP had identified another 2,625 positive persons (2,040 tested and 585 documented) bringing the total number of persons who knew their status to 13,328 and the percent of HIV-positive persons knowing their status to 93% (13,328/14720) of the estimated HIV-positive persons in the 15 communities. The 95% CI (13,497, 15,041) would place the coverage of knowledge of status between 89 and 99%. BCPP identified 98% of the estimated number of HIV positive women in the intervention communities (8,981/9,167) and 85% of the estimated number of HIV positive men (5,103/4,347).

**Fig 4:**
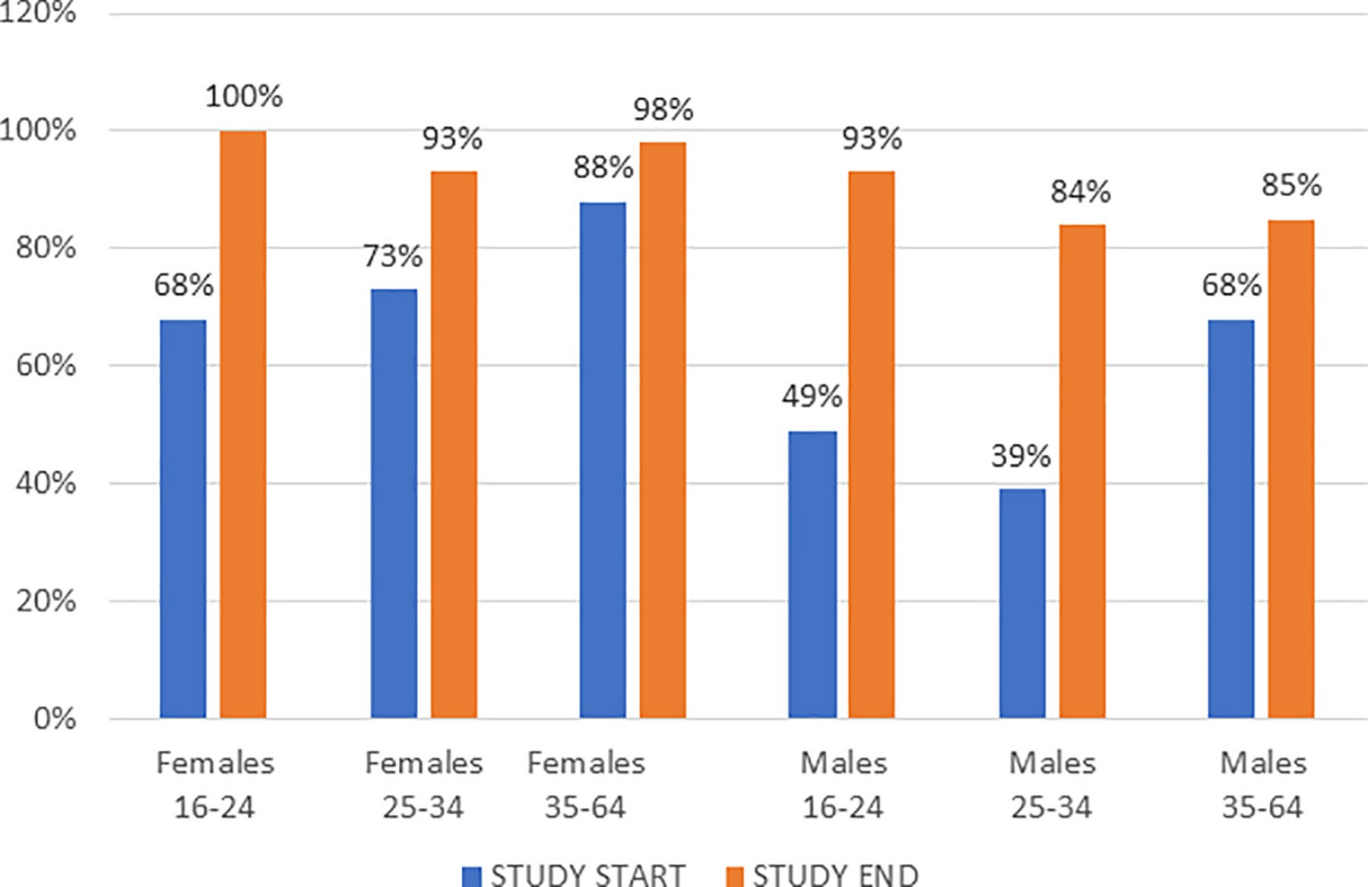
Increases in population-level knowledge of HIV-positive status from study start to study end in the intervention communities by sex and age.

Increases in knowledge of HIV-positive status from study start to study end varied by age and sex. Before the study began, 7,527 women knew their status which increased by 19% to 8,981 at study end. Among men, 3,176 knew their status at baseline which increased to 4,347 or by 37% at study end. Greater gains in knowledge of HIV-positive status were made by younger persons 16–24, both male (89%) and female (73%), and by men 25–34 (114%) compared to women and men 35–64 (27% and 24%, respectively) (Fig 4).

## Discussion

A primary goal of BCPP was to increase knowledge of status among HIV-positive persons in the intervention communities through community-based testing activities. By study end, 93% of the estimated population of HIV-positive persons in the 15 intervention communities knew their HIV status. Knowledge of status increased by 25% over the course of the study with greatest increases seen among men and youth who had lower rates of knowledge of positive status before the study was initiated. These findings, consistent with published literature from other combination prevention studies in the region, demonstrate that achievement of the 1^st^ UNAIDS goal of 90% of positive persons knowing their status is feasible with comprehensive testing approaches. Although Botswana had high coverage of HIV testing at study start, a substantial number of people had been missed by the long-standing national testing program. We were able to reach undiagnosed persons by implementing a multi-component testing program, increasing coverage among men by 37% and youth by 77%. This increase in the percentage of people who knew their positive status and their linkage to treatment may have substantially contributed to the 30% reduction in new HIV infections found in BCPP in the intervention compared to control communities [21]. Additionally, 1,617 previously diagnosed HIV-positive persons not on ART were identified. Uptake of treatment by these persons may also have contributed to the reduction in incidence.

In BCPP, different testing modalities reached different sub-groups of the population. A higher percentage of women were reached in the home and a higher percentage of men and youth were each reached through mobile settings. We enhanced the participation of men and young people in HIV testing by targeting them through mobile outreach to workplaces, including construction sites, farms, cattle posts, bars/shebeens, and mobilizing them through sporting events. A number of interventions to increase men’s testing in sub-Saharan Africa have been described elsewhere [2, 8, 9, 16–18]. Our experience suggests that testing programs should broaden the strategies and venues for finding men and youth and take testing services to places they frequent rather than waiting for them to access services.

Despite the fact that more men were accessed through targeted mobile testing than home-based testing, the percentage of new positives found among men over 25 years of age in BCPP was higher in the home than in mobile settings. For youth and older women, the yield of new positives did not differ for home and mobile testing. Based on these results, we believe that both home and mobile testing services should be considered for reaching undiagnosed HIV-positive persons across all age and sex strata. A previous study which employed only home-based testing in Botswana missed an estimated 48.5% of men due to mobility and migration [19]. These findings have led some testing implementers to discard home-based testing as a venue for finding men. Home-based testing in BCPP, however, was enhanced by counselors visiting the home after work hours, on the weekends, on holidays, making multiple visits to the home, and making appointments convenient to the participant. These enhancements appear necessary for an effective home-based testing program. Although home-based testing has been found to be more expensive to implement than mobile testing [7, 22], it may be a cost-effective testing strategy given the high yield of positive men who had not been previously diagnosed.

As coverage of HIV testing improves in countries, case finding becomes increasingly difficult and potentially more costly as more community approaches are needed to find harder to reach populations who don’t access health facilities [23, 24]. The costs of the home-based and mobile testing modalities used in this study were reported previously [22] and are comparable to the costs of these activities in other southern African middle-income countries. While some community-based testing activities are more costly than facility-based testing depending upon the country, setting and intensity of the activity, many studies have shown that community-based testing activities are comparable in costs to facility-testing [7,8]. Community-based testing activities have been shown repeatedly to be effective case finding approaches for reaching those populations who don’t access health facilities and for achieving high coverage levels [7,8]. With program budgets being flatlined or decreasing, countries and programs are being pressured to be more targeted in their testing efforts. These approaches should be considered part of an optimal HIV testing portfolio targeting those sub-populations hardest to reach (e.g., men, young people, key populations) programmed within the context of the country. Targeting these approaches such as testing partners of newly diagnosed HIV-positive persons in the home if they do not come to the facility rather than untargeted door-to-door testing, offering mobile services in areas where HIV prevalence and population are highest, etc. may be needed if resources are limited and may be more efficient approaches to finding undiagnosed HIV-positive persons. Further research is needed on these targeted approaches and cost comparisons.

The proportion of HIV-positive persons was significantly higher among women compared to men, with young women (16–24 and 25–34) nearly three times more likely to be infected than males in the same age groups. This finding is consistent with the trends in sub-Saharan Africa and findings from other studies [4, 6, 12, 15, 19, 20, 25–28]. Furthermore, HIV positivity peaked at a younger age for women (35–44 years) when nearly half the women were HIV-infected, while for men, HIV positivity peaked at an older age (45–55) at 40%. UNAIDS estimates that 3 out of 4 new HIV infections in sub-Saharan Africa are among girls and young women aged 15–24 years who are two to three times more likely to be living with HIV than men [6, 26]. Several factors have been found to contribute to the vulnerability of young women to HIV infection, including the complexities of transition to adulthood, partnering with older men, and biological factors [26]. Several studies have suggested that low testing rates, late HIV diagnosis, low linkage to care, and low ART initiation among men explain in part the persistently high HIV incidence among young women in sub-Saharan Africa [16]. Our findings and existing literature suggest that intensified efforts to reach men over 25 years with testing, linkage to care, and treatment are urgently needed for the health of men and because untreated infected men may be fueling new HIV infections among young women [6, 19, 26, 28]. BCPP showed that men and youth can be reached and are willing to learn their HIV status. With the multi-pronged testing approach, significant gains in knowledge of positive HIV status were made among both men and youth.

BCPP had several limitations. First, mobility of the population may have reduced our ability to find all HIV-positive persons; some residents were absent despite several attempts to reach them through home visits. In addition, we were not able to link all mobile testers to specific households to determine how many of those not assessed in homes were actually tested through mobile activities. Also, in- and out-migration in the community was not measured during the course of the study. Second, the calculated population estimates of those living in the communities did not account for changes in overall population numbers from study start to study end. Population estimates also had to extrapolate for the households which appeared to be residential and habitable but were never enumerated. Thus, the estimation of HIV-positive persons living in these communities might have resulted in under or overestimation of coverage of knowledge of status as the exact numbers are not known. Third, only rural and peri-urban communities were included in the study which may not be generalizable to more urban communities; urban communities not included might have had different coverage or required different testing strategies. Finally, community-based testing, and especially home-based testing, are resource-intensive interventions. As pressures increase to reduce program costs and implement higher yield and lower cost strategies, countries and programs will have to consider where these effective but potentially more costly community-based models fit into their portfolio for reaching the 1st 90 or 95, especially with men, youth, and key populations.

In conclusion, different HIV testing strategies are needed to target different subpopulations in settings where testing coverage is already high. In this study, each modality contributed to identification of new positives. Program implementers need to consider context and setting in the development of strategies to increase testing coverage in their countries. Our findings add to existing literature and demonstrate that barriers to HIV testing can be overcome, and the 1^st^ 90 target can be achieved through a multi-modality testing strategy. Up to date data are needed to inform effective implementation strategies for reaching persons who have been missed by programmatic testing efforts.

## Supporting information

S1 File BCPP protocol. (DOCX)

S2 File Trend checklist. (PDF)

## References

[1] UNAIDS. Global HIV & AIDS statistics—2018 fact sheet. Geneva, Switzerland: UNAIDS; 2018.

[2] Petersen M, Balzer L, Kwarisiima D, Sang N, Chamie G, Ayieko J, et al Association of implementation of a universal testing and treatment intervention with HIV diagnosis, receipt of antirotroviral therapy, and viral suppression in East Africa. JAMA. 2017;317(21):2196–2206. 10.1001/jama.2017.5705 28586888 PMC5734234

[3] Iwuji CC, Orne-Gliemann J, Larmarange J, Balestre E, Thiebaut R, Tanser F, et al Universal test and treat and the HIV epidemic in rural South Africa: A phase 4, open-label, community cluster randomised trial. Lancet HIV. 2018;5:e116–25. 10.1016/S2352-3018(17)30205-9 29199100

[4] Barnabas R, van Rooyen H, Tumwesigye E, Brantley J, Baeten J, van Heerden A, et al Uptake of antiretroviral therapy and male circumcision after community-based testing and strategies for linkage to care versus standard clinic referral: a multisite, open-label, randomised controlled trial in South Africa and Uganda. Lancet HIV. 2016;3:e212–20. 10.1016/S2352-3018(16)00020-5 27126488 PMC4852382

[5] Iwuji CC, Orne-Gliemann J, Larmarange L, Okesola N, Tanser F, Thiebaut R. et al Uptake of home-based HIV testing, linkage to care, and community attitudes about ART in rural KwaZulu-Natal, South Africa: Descriptive results from the first phase of ANRS 12249 TasP cluster-randomised trial. PLoS Med. 2016;13(8):e1002117 10.1371/journal.pmed.1002117 27504637 PMC4978506

[6] UNAIDS. Ending AIDS: Progress towards the 90-90-90. Geneva, Switzerland: UNAIDS; 2017.

[7] Sharma M, Ying R, Tarr G, Barnabas R. Systematic review and meta-analysis of community and facility-based HIV testing to address linkage to care gaps in sub-Saharan Africa. Nature. 2015;528:S77–85. 10.1038/nature16044 26633769 PMC4778960

[8] Suthar A, Ford N, Bachanas P, Wong V, Rajan J, Saltzman A, et al Towards universal voluntary HIV testing and counselling: A systematic review and meta-analysis of community-based approaches. PLOS Medicine. 2013;10(8):e1001496 10.1371/journal.pmed.1001496 23966838 PMC3742447

[9] Hensen B, Taoka S, Lewis JJ, Weiss HA, Hargreaves J. Systematic review of strategies to increase men’s HIV-testing in Sub-Saharan Africa. AIDS. 2014;28:2133–45. 10.1097/QAD.0000000000000395 25062091 PMC4819892

[10] Shanuabe K, Schaap A, Floyd S, Phiri M, Griffith S, Chaila J, et al What works–reaching universal HIV testing: lessons from the HPTN 071 (PopART) trial in Zambia. AIDS. 2017;31:1555–64. 10.1097/QAD.0000000000001514 28471766 PMC5491236

[11] Hayes R, Floyd S, Schaap A, Shanuabe K, Bock P, Sabapathy K, et al A universal testing and treatment intervention to improve HIV control: One-year results from intervention communities in Zambia in the HPTN 071 (PopART) cluster -randomised trial. PLoS Med. 2017;14(5):e1002292 10.1371/journal.pmed.1002292 28464041 PMC5412988

[12] Gaolathe T, Wirth KE, Holme MP, Makhema J, Moyo S, Chakalisa U, et al Botswana’s progress toward achieving the 2020 UNAIDS 90-90-90 antiretroviral therapy and virologic suppression goals: A population-based survey. Lancet HIV. 2016;3(5):e221–30. 10.1016/S2352-3018(16)00037-0 27126489 PMC5146754

[13] Maman D, Chilima B, Masiku C, Ayouda A, Masson S, Szumilin E, et al Closer to 90-90-90. The cascade of care after 10 years of ART scale-up in rural Malawi: a population study. JIAS. 2016;19:20673. 10.7448/IAS.19.1.20673 PMC4760102 26894388

[14] Barnabas R, van Rooyen H, Tumwesigye E, Murnane P, Baeten J, Humphries H, et al Initiation of antiretroviral therapy and viral suppression after home HIV testing in KwaZulu-Natal, South Africa, and Mbarara district, Uganda. Lancet HIV. 2014;1(2):e68–76. 10.1016/S2352-3018(14)70024-4 25601912 PMC4292844

[15] van Rooyen H, Barnabas R, Baeten J, Phakathi Z, Joseph P, Krows M, et al High HIV testing uptake and linkage to care in a novel program of home-based HIV counseling and testing with facilitated referral in KwaZulu-Natal, South Africa. J Acquir Immune Defic Syndr. 2013;64(1):e1–8. 10.1097/QAI.0b013e31829b567d 23714740 PMC3744613

[16] Chamie G, Clark TD, Kabami J, Kadede K, Ssemondo E, Steinfeld R, et al A hybrid mobile HIV testing approach for population wide HIV testing in rural East Africa: An observational study. Lancet HIV. 2016;3(3):e111–19. 10.1016/S2352-3018(15)00251-9 26939734 PMC4780220

[17] Chamie G, Kwarisiima D, Clark TD, Kabami J, Jain V, Geng E, et al Uptake of community HIV testing during a multi-disease health campaign in rural Uganda. PLoS ONE. 2014;9(1):e84317 10.1371/journal.pone.0084317 24392124 PMC3879307

[18] Sabapathy K, Van den Bergh R, Fidler S, Hayes R, Ford N. Uptake of home-based voluntary HIV testing in Sub-Saharan Africa: A systematic review and meta-analysis. PLoS Med. 2012;9(12): e1001351 10.1371/journal.pmed.1001351 23226107 PMC3514284

[19] Novitsky V, Bussman H, Okui L, Logan A, Moyo S, van Widenfelt E, et al Estimated age and gender profile of individuals missed by a home-based HIV testing and counseling campiagn in a Botswana community. J. Int. AIDS Soc. 2015;18:19918 10.7448/IAS.18.1.19918 26028155 PMC4450241

[20] Botswana Statistics. Botswana AIDS Impact Survey (BAIS) IV. Gaborone, Botswana: Statistics Botswana; 2013.

[21] Makhema J, Wirth K, Pretorius Holme M, Gaolathe T, Mmalane M, Kadima E, et al Universal Testing, Expanded Treatment, and Incidence of HIV Infection in Botswana. N Eng J Med. 2019 Jul 18;381(3):230–42. 10.1056/NEJMoa1812281 PMC6800102 31314967

[22] Lasry A, Bachanas P, Suraratdecha C, Alwano MG, Behel S, Pals S, et al Cost of community-based HIV testing activities to reach saturation in Botswana. AIDS Behav. 2019;23(4):875–82. 10.1007/s10461-019-02408-9 30673897 PMC6782058

[23] UNAIDS. The Gap Report. Geneva, Switzerland: UNAIDS; 2015.

[24] WHO. Consolidated Guidelines on HIV Testing Services. Geneva: World Health Organization; 2015. 26378328

[25] Shanuabe K, Schaap A, Chaila M.J, Floyd S, Mackworth-Young C, Hoddinott G, et al Community intervention improves knowledge of HIV status of adolescents in Zambia: Findings from HPTN 071 –PoPART for Youth Study. AIDS. 2017;31(suppl.3):S221–32. 28665880 10.1097/QAD.0000000000001530 PMC5497780

[26] Karim Q, Baxter C, and Birx D. Prevention of HIV in adolescent girls and young women: Key to an AIDS-Free Generation. J Acquir Immune Defic Syndr. 2017;75:S17–26. 10.1097/QAI.0000000000001316 28398993

[27] Choko A, Desmond N, Webb E, Chavula K, Napierala-Mavedzenge S, Gaydos C, et al The uptake and accuracy of oral kits for HIV self-testing in high HIV prevalence settings: A cross-sectional feasibility study in Blantyre, Malawi. PLOS Med. 2011;8(10):e1001102 10.1371/journal.pmed.1001102 21990966 PMC3186813

[28] Kadede K, Ruel T, Kabami J, Ssemondo E, Sang N, Kwarisiima D, et al Increased adolescent HIV testing with a hybrid mobile strategy in Uganda and Kenya. AIDS. 2016;30(14):2121–26. 10.1097/QAD.0000000000001180 27258399 PMC5007167

