# Supplementary material for: Increasing knowledge of HIV status in a country with high HIV testing coverage: Results from the Botswana Combination Prevention Project
Source: PLoS One. 2019 Nov 25;14(11):e0225076. doi: 10.1371/journal.pone.0225076 (PMC6876886; doi:10.1371/journal.pone.0225076)
Supplement: S1 File — (DOCX) [file pone.0225076.s001.docx]

**Botswana Combination Prevention Project**

**(BCPP)**

**Intervention Protocol**

**(Protocol #3)**

__________________________________________

**Implementation, Monitoring, and Evaluation of a Combination of HIV Prevention Interventions in Rural and Peri-Urban Communities in Botswana**

**Version 6.0**

**September 22, 2017**

**Collaborating Institutions:**

Botswana Ministry of Health (MOH)

Harvard T. H. Chan School of Public Health (Harvard Chan School)

U.S. Centers for Disease Control and Prevention (CDC)

**Funded by**:

The United States President’s Emergency Plan for AIDS Relief (PEPFAR) through

United States Centers for Disease Control and Prevention (CDC)

**PRINCIPAL INVESTIGATORS:**

**Janet Moore, PhD**

Branch Chief, HIV Prevention Branch

Division of Global HIV/AIDS (DGHA), Center for Global Health (CGH)

United States Centers for Disease Control and Prevention (CDC)

Address: 1600 Clifton Road, Atlanta, GA, 30333, U.S.A

Email: jym3@cdc.gov Office Phone: 404.639.8954

**Refeletswe Lebelonyane, MD**

Combination Prevention Coordinator, MOH

Ministry of Health, Botswana Government

Enclave Plot No. 54609

Gaborone, Botswana

Email:  rlebelonyane@gov.bw

**Shenaaz El Halabi, (PS) MPH**

Permanent Secretary

Botswana Ministry of Health

Address: Private Bag 0038, Gaborone, Botswana

Email: sel-halabi@gov.bw Office Phone: +267-363-2069

**Pamela Bachanas, PhD**

Behavioral Scientist, HIV Prevention Branch

Division of Global HIV/AIDS (DGHA), Center for Global Health (CGH)

United States Centers for Disease Control and Prevention (CDC)

Address: 1600 Clifton Road, Atlanta, GA, 30333, U.S.A

Email: ddt6@cdc.gov Office Phone: 404.639.8110

**ROLES OF INSTITUTIONS AND PRINCIPAL INVESTIGATORS**

**Institutions**: Close coordination between MOH, CDC, HSPH, BHP, and other implementing partners is needed to ensure seamless implementation of research and program activities. MOH and CDC will lead and ultimately be responsible the Intervention Protocol and HSPH and BHP will lead the Evaluation Protocol.

**Principal Investigators**:

**Janet Moore/ Pamela Bachanas:** CDC (PIs) responsible for preparation and design of the study protocol, monitoring of study progress, creation and monitoring of research budgets, and preparation of monthly progress reports.

**Refeletswe Lebelonyane/ Shenaaz El Halabi:** MOH Co-Principal Investigator (Co-PI) responsible for protocol review and in-country implementation oversight.

**CO-INVESTIGATOR LISTS BY INSTITUTION**

**Botswana Ministry of Health (MOH), Gaborone, Botswana**

| **Name** | **Role** | **Contact Email** |
| --- | --- | --- |

Ms. Dinah Ramaabya (Nursing) ARV Program dramaabya@gov.bw

Ms. Chipo Phetlo (Nursing) PMTCT cphetlo@gov.bw

Mr. S. Makathaye, Dip Health Educ. BCIC smalathaye@gov.bw

Mr. C. Ntsuape, BEd (Nursing Educ) SMC ontsuape@gov.bw

Ms. Nokuthula Manjingo (Nursing) HIV Testing & Counselling [mmajingo@gov.bw](mailto:mmajingo@gov.bw)

Dr. Joe Theu ((Medical Officer) Clinical Care jtheu@gov.bw

**Centers for Disease Control and Prevention (CDC), Atlanta, GA, USA, and Gaborone, Botswana**

| **Name** | **Role** | | **Contact Email** |
| --- | --- | --- | --- |
| Elliot Raizes, MD | Clinical Consultant | | gwq0@cdc.gov |
| Ebi Bile, BSc. MA, DU | In-country Lab Oversight | | efb6@bw.cdc.gov |
| Peter Fonjungo, PhD | HQ Lab Oversight | | pdf4@cdc.gov |
| Sherri Pals, PhD | Statistics | | sfv3@cdc.gov |
| Chunfu Yang, PhD | Laboratory | | cxy0@cdc.gov |
| Mary Grace Alwano | In Country HTC/ LTC Project Coordinator | mea9@bw.cdc.gov | |
| Stephanie Behel, MPH | HTC and LTC | zmd7@cdc.gov | |
| Naomi Bock, MD | Voluntary Medical MC | neb2@cdc.gov | |
| Stephanie Davis, MD, MPH | Voluntary Medical MC | vic6@cdc.gov | |
| Lisa Block, MS | Project Manager | kwk5@cdc.gov | |
| Lisa Mills, MD  William Abrams | Co-investigator  Program Administration | Ioj7@cdc.gov  hvf8@cdc.gov | |
| Arielle Lasry PhD | HTC Costing | [ftn9@cdc.gov](mailto:ftn9@cdc.gov) | |

**Harvard T. H. Chan School of Public Health, Boston, MA, USA**

| **Name** | **Role** | **Contact Email** |
| --- | --- | --- |
| Myron Essex, DVM, PhD | Co-Investigator | [messex@hsph.harvard.edu](mailto:messex@hsph.harvard.edu) |
| Eric Tchetgen, PhD | Statistics and Epidemiology | etchetge@hsph.harvard.edu |
| Scott Dryden-Peterson, MD | Treatment and PMTCT | [scott_peterson@post.harvard.edu](mailto:scott_peterson@post.harvard.edu) |
| Shahin Lockman, MD, MSc | Treatment and PMTCT | [slockman@hsph.harvard.edu](mailto:slockman@hsph.harvard.edu) |
| Rebeca Plank, MD | SMC | [rplank@partners.org](mailto:rplank@partners.org) |
| Molly Pretorius Holme, MSc | Research operations | [mpretori@hsph.harvard.edu](mailto:mpretori@hsph.harvard.edu) |
| Roger Shapiro, MD, MPH | Treatment and PMTCT | rshapiro@hsph.harvard.edu |
| Kate Powis, MD, MPH | Research Operations, Treatment and PMTCT | kpowis@mgh.harvard.edu |

**Botswana–Harvard AIDS Institute Partnership (BHP), Gaborone, Botswana**

| **Name** | **Role** | **Contact Email** |
| --- | --- | --- |
| Tendani Gaolathe, MD | Treatment and PMTCT,  Field research operations | [tgaolathe@bhp.org.bw](mailto:tgaolathe@bhp.org.bw) |
| Joseph Makhema, MD | BHP research operations | [jmakhema@bhp.org.bw](mailto:jmakhema@bhp.org.bw) |
| Sikhulile Moyo, BSc, MSc, MPH | Lab | [smoyo@bhp.org.bw](mailto:smoyo@bhp.org.bw) |
| Mompati Mmalane, MD, FRCS-Ed, MSc | Community mobilization and research operations | mmmalane@bhp.org.bw |
| Erik van Widenfelt, BA | Research data systems | [ewidenfelt@bhp.org.bw](mailto:ewidenfelt@bhp.org.bw) |

**TABLE OF CONTENTS**

[Principal Investigators](#_Toc267057751) 2

[List of Co-Investigators](#_Toc267057751) 3

[Table of Contents](#_Toc267057747) 5

[List of Tables and Figures](#_Toc267057747) 7

[List of Acronyms and Abbreviations](#_Toc267057750) 8

[Intervention Protocol Synopisis](#_Toc267057750) 10

[1.0 INTRODUCTION](#_Toc267057753) 12

[1.1 Epidemic in Botswana](#_Toc267057754)

[1.2 Combination Prevention Interventions](#_Toc267057757)

1.3 Antiretroviral Treatment as a Component of the Combination Prevention Package

[1.4 Proposed Combination Prevention (CP) Package](#_Toc267057761)

[1.5 Relationship Between Intervention Study Design and Impact Evaluation](#_Toc267057761)

[2.0 STUDY OBJECTIVES](#_Toc267057764) 16

[3.0 OVERVIEW OF STUDY METHODS](#_Toc267057767) 16

[4.0 STUDY POPULATIONS](#_Toc267057772) 16

[4.1 Selection of Study Communities](#_Toc267057768)

[4.2 Matching of Study Communities](#_Toc267057769)

[4.3 Eligibility of Community Members](#_Toc267057770)

[4.4](#_Toc267057771) Enhancement of Services in all Study Communities

[4.5](#_Toc267057771)  Eligibility Criteria for Inclusion in Interventions in CP Communities

4.6 Rationale for Exclusion in Interventions in CP Communities

**5.0 SAMPLE SIZE AND SAMPLE SELECTION METHODS…………………………………………………………….21**

[6.0 INTERVENTION PROCEDURES](#_Toc267057772) 22

6.1 Overview of Intervention Strategy

6.2 Stakeholder Involvement, Community Engagement, and Community Mobilization

6.3 HIV Testing and Counseling (HTC)

6.4 Linkage to HIV Care and Treatment

6.5 Male Circumcision (MC)

6.6 Identification, Linkage, and Treatment of HIV+ Pregnant Women

6.7 HIV Care and Treatment

[7.0 LABORATORY METHODS](#_Toc267057787) 32

7.1 Overview of Laboratory Methods

[7.2 Training of Laboratory Personnel](#_Toc267057788)

[7.3 Specimen Collection, Transport, and Processing](#_Toc267057789)

[7.4](#_Toc267057789)  Laboratory Testing for HTC Campaigns

[7.5](#_Toc267057789)  Laboratory Testing for IDCC Patients

[7.6 Quality Assurance](#_Toc267057789)

[7.7 Biosafety and Waste Management](#_Toc267057789)

[7.8 Specimen Storage and Shipment](#_Toc267057789)

[8.0 DATA SYSTEMS AND MANAGEMENT](#_Toc267057790) ………35

[8.1 Data Collection, Capture, and Flow](#_Toc267057791)

[8.2](#_Toc267057792) Data Security

[8.3](#_Toc267057793) Data Quality

[8.4](#_Toc267057794) Confidentiality of data

[9.0](#_Toc267057799) DATA AND ANALYSIS PLANS…………………………………………………………………………………………….40

[9.1 HIV Testing and Counseling and Linkage to Care](#_Toc267057791)

[9.2 Care and Treatment](#_Toc267057792)

[9.3 PMTCT Services](#_Toc267057793)

[9.4](#_Toc267057794) Male Circumcision

[9.5](#_Toc267057794) Analysis Plans

[10.0 ETHICAL CONSIDERATIONS…](#_Toc267057809) 5

[10.1](#_Toc267057810)  Ethical Review

[10.2](#_Toc267057811)  Informed Consent

[10.3](#_Toc267057812)  Risks

[10.4](#_Toc267057812)  Benefits of Research Participation

10.5 Confidentiality

[10.6](#_Toc267057812)  Protocol Compliance and Compliance with UN Convention on the Rights of the Child

[10.7](#_Toc267057812)  Future Use of Stored Samples

[11.0 STUDY SAFETY AND MONITORING PLAN](#_Toc267057813) 49

[11.1](#_Toc267057810)  Reporting Adverse Events

[11.2](#_Toc267057811)  Data Safety and Monitoring Board (DSMB)

[11.3](#_Toc267057812)  Protocol Violations

[11.4](#_Toc267057812)  Monitoring Plan

[12.0 ADMINISTRATIVE PROCEDURES AND INFORMATION DISSEMINATION](#_Toc267057813) 52

[12.1](#_Toc267057811)  [Study Records and Document Retention](#ADM2)

[12.2](#_Toc267057812)  Information Dissemination and Publication Plan

[13.0 REFERENCES](#_Toc267057813) 53

APPENDICES:

_________________________________________________________________________________

Cross-Protocol Appendices:

Appendix C2 A. List of Study Communities and Matching Criteria for Community Pairs

B. Back-up Communities in Selected 30 Communities Decline, unavailable or deemed unsuitable for study inclusion

Figure A: Map of Study Communities in Three Regions

Figure B: Map of Study Communities in the Southern Region

Figure C: Map of Study Communities in the Central Region

Figure D: Map of Study Communities in the Northern Region

Appendix C3 Size of Study Populations

Appendix C4 Data Safety Monitoring Plan

Protocol 3 Appendix:

Appendix P1 Summary of Variables to be Collected for Ya Tsie Protocol 3

_________________________________________________________________________________

LIST OF TABLES AND FIGURES

**Figure 1:** Data Flow for Combination Prevention Communities

**Table 1:** Precision for 95% confidence intervals for uptake of CP interventions in CP communities

**Table 2:** BCPP Key Indicators

**Table 3**: Summary of approach to consent for various components of the study

**ACRONYMS AND ABBREVIATIONS**

AE adverse event

AIDS acquired immunodeficiency syndrome

AHS annual household surveys (follow-up visits of the BHS and HIC)

ANC antenatal clinic (or care)

ART antiretroviral treatment (or therapy)

ARV antiretroviral (drug)

BCPP Botswana Combination Prevention Project

BHHRL Botswana–Harvard HIV Reference Laboratory

BHP Botswana–Harvard AIDS Institute Partnership

BHS baseline household survey

CDC Centers for Disease Control and Prevention

CI confidence interval

CP combination prevention

CPC combination prevention community

CRT community-randomized trial

DSMB Data Safety Monitoring Board

ECC enhanced care communities

ELISA enzyme-linked immunosorbent assay

ESS end of study survey

ETC expanded treatment cohort

HIC HIV incidence cohort

HIV human immunodeficiency virus

HIV-1 RNA HIV-1 RNA load in plasma, viral load

HIVDR HIV drug resistance

HTC HIV testing and counseling

HVL high viral load

IAS International AIDS Society

IDCC infectious disease care clinic

IPMS integrated patient management system

IRB institutional review board

LFT liver function test

LTC linkage-to-care

MC male circumcision

M&E monitoring and evaluation

MOH Ministry of Health

MTCT mother-to-child (HIV) transmission

OHRP Office for Human Research Protections

OI opportunistic infection

Option B+ indefinite continuation of ART for women who start ART for PMTCT

PEP post-exposure prophylaxis

PEPFAR President’s Emergency Plan for AIDS Relief

PII personally identifiable information

PIMS patient information management system

PMTCT prevention of mother-to-child (HIV) transmission

PLHIV people living with HIV

POC point-of-care

POB pregnant/pregnancy or breastfeeding

QA quality assurance

QC quality control

RHT Routine HIV testing

SAE serious adverse event

SD standard deviation

SMC safe male circumcision (Botswana government initiative)

SMS short message service

SOC standard of care

SoE schedule of events

SOP standard operating procedure

study arm a group of 15 communities that is randomized to receive either standard of care or study interventions with respect to HIV prevention measures

TB tuberculosis

UN United Nations

UNAIDS Joint United Nations Program on HIV/AIDS

UTT Universal Test and Treat

VDC village development committee

VF virologic failure

VL viral load

VPN virtual private network

WHO World Health Organization

**BCPP Intervention Protocol (#3)**

**Protocol Synopsis**

**Overview:** The BCPP study is composed of 2 active interlocking protocols, the Evaluation Protocol (1) and the Intervention Protocol (3). The Closed Clinical Cohort Protocol (Protocol 2) was withdrawn in 2015. An overview of the overarching BCPP study is provided in the BCPP Overall Schema located in the Evaluation Protocol.

**Primary Objective of Protocol 3**

1. To implement the combination prevention (CP) intervention package in combination prevention communities (CPC) and describe uptake of these interventions in CPCs (expanded HIV Testing and Counseling (HTC), strengthened Male Circumcision (MC), expanded HIV Care and Treatment, and strengthened PMTCT services).

**Secondary Objectives of Protocol 3**

1. To describe retention in the clinical cascade of care from HIV diagnosis to viral suppression among all HIV-infected persons.
2. To describe uptake of expanded antiretroviral treatment (ART) and clinical outcomes including viral suppression among HIV-positive persons receiving expanded ART.
3. To examine demographic, clinical, and social/behavioral facilitators and barriers to uptake of CP interventions.
4. To examine demographic factors, including community of residence, that affect access to health care services.

**Study Design:**

The BCPP study is a pair-matched community-randomized trial. This protocol describes the programmatic interventions, including expanded ART, and program monitoring activities that will be conducted as part of the larger BCPP project.

**Study Population**:

Thirty community clusters were selected from three geographic regions in Botswana. Pre-randomization, community pairs were matched on community characteristics thought to be associated with the outcome of interest of the BCPP (HIV incidence) to improve balance between arms and statistical efficiency. The average total population size of each of the targeted communities is 6,000 (range 2,400 - 12,000), giving a total population across all 30 communities of about 180,800, of whom about 105,000 are adults aged 16-64.

**Study Procedures:**

Matched community pairs (15 communities per arm) were randomized 1:1 to Enhanced Care (ECC; Comparison) or Combination Prevention (CPC; Intervention) arms prior to the baseline household survey (BHS) that is conducted as part of the BCPP Evaluation Protocol. Initiation of intervention activities in the CPCs begin immediately following completion of enrollment in the BHS and continue for the duration of the study. CPCs receive the combination prevention intervention package, which includes strengthened HTC, MC linkage, PMTCT services, and expanded HIV care and treatment for HIV-infected persons 16-64 years. In addition, expanded ART for existing HIV-infected community clinic patients will be offered in the CPCs. Routinely collected clinical data will be used to assess the clinical outcomes for all HIV positive patients.

**Study Duration:** All study activities will occur over a period of approximately 60 months.

1. **INTRODUCTION AND BACKGROUND**

**1.1** **Epidemic in Botswana**

Thirty years into the global HIV pandemic, HIV prevalence and incidence remain high in sub-Saharan Africa. Although this region represents only 12% of the world’s population, it accounts for approximately 67% of the world’s 34 million HIV-infected persons, and annually, about 70% of the world’s 2.7 million new infections occur in this region ([UNAIDS 2010](#_ENREF_30)). Despite substantial progress in providing HIV treatment and prevention of mother-to-child transmission services (PMTCT) to its citizens, an estimated 25% of adult residents of Botswana are HIV-infected, the second highest adult HIV prevalence in the world, and about 14,000 persons are newly infected with HIV annually, the third highest national HIV incidence rate globally ([UNAIDS 2010](#_ENREF_30)).

**1.2 Combination Prevention Interventions**

In the past five years several HIV prevention interventions have proven to be highly effective at reducing transmission of HIV and have offered hope for containing and possibly reversing epidemics in sub-Saharan Africa. For HIV-negative men, MC has been shown to significantly reduce HIV acquisition from HIV-infected female partners. Three randomized controlled trials of circumcision, involving more than 10,000 men in sub-Saharan Africa, demonstrated a 60% reduction in HIV acquisition risk ([Auvert, Taljaard et al. 2005](#_ENREF_3), [Bailey, Moses et al. 2007](#_ENREF_4), [Gray, Kigozi et al. 2007](#_ENREF_27)). Additionally, antiretroviral treatment (ART) of HIV-infected persons has been shown to reduce viral load (VL) and consequently the risk of HIV transmission to uninfected partners in sero-discordant couples by as much as 96% ([Cohen, Chen et al. 2011](#_ENREF_10)). Similarly, the use of antiretrovirals to prevent transmission of HIV from infected mothers to their infants during pregnancy, birth, and breastfeeding can reduce transmission to below 5% ([Mahy, Stover et al. 2010](#_ENREF_47); Shapiro, Hughs et al. 2013).

Despite considerable success in scaling up these interventions in many sub-Saharan African countries, especially those supported by the U.S. President’s Emergency Plan for AIDS Relief (PEPFAR), adult HIV incidence has remained high (>1%) in many regions in Southern Africa (Botswana, Swaziland, Lesotho, Kwa-Zulu Natal in South Africa, and Southern Mozambique). Multiple factors may account for high HIV incidence in this part of the world. Firstly, viral characteristics of HIV-1 subtype C, endemic in Southern Africa, may make it more infectious than other viral subtypes ([Novitsky, Wang et al. 2010](#_ENREF_57), [Novitsky, Ndung'u et al. 2011](#_ENREF_56)). For example, recent evidence suggests that about one third of persons infected with HIV-1 sub-type C, maintain very high viral loads (VL>50,000 copies/ml) for more than 6 months after sero-conversion. In contrast, viral load following HIV-1 subtype B infection (the prevalent subtype in the United States and Europe) resolves to a steady-state viral set point below 50,000 copies/ml in almost all individuals within four to six months ([Daar, Moudgil et al. 1991](#_ENREF_14), [Mellors, Munoz et al. 1997](#_ENREF_49), [Kaufmann, Cunningham et al. 1998](#_ENREF_39), [Little, McLean et al. 1999](#_ENREF_45)).

Secondly, the scale and quality of the HIV prevention interventions may have been insufficient to reduce HIV incidence among adults in Southern Africa to levels observed in other parts of the world. For example, the main mechanism by which scale-up of HTC and antiretroviral therapy reduces HIV transmission risk in the community, is by reducing viral load in resident HIV-infected persons, ideally to undetectable levels (VL<400 copies/ml). Studies have suggested that every 0.5 log reduction in an individual’s HIV-1 viral load is equivalent to a 40% reduction in individual HIV transmission risk ([Modjarrad, Chamot et al. 2008](#_ENREF_53)), and once the viral load is fully suppressed (VL <400 copies/ml) individual HIV transmission risk is virtually zero ([Attia, Egger et al. 2009](#_ENREF_1)). Despite considerable scale-up of access to HTC and ART, poor retention of HIV-infected persons in the HIV care and treatment cascade from diagnosis to ART follow-up ([Rosen and Fox 2011](#_ENREF_61)), has mitigated the ability of HTC and HIV care and treatment programs in Southern Africa to reduce the prevalence of HIV-infected persons with detectable viral loads (VL>400 copies/ml). In a meta-analysis of pre-ART retention in Africa, Rosen et al. reported that only 59% of newly diagnosed adults are successfully linked to HIV care and treatment services, only 46% are retained from initial registration and staging at the facility to ART-eligibility, and only 68% are retained from ART eligibility to ART initiation ([Rosen and Fox 2011](#_ENREF_61)). Of those adults who initiate ART, ~30% are no longer on therapy by 3 years of follow-up, with most of those no longer in care considered lost to follow-up ([Fox and Rosen 2010](#_ENREF_20)). Therefore, even if 50% of HIV-infected adults in Botswana know their HIV status ([BAIS 2008](#_ENREF_5)), a considerably lower proportion have a suppressed viral load ([Gardner, McLees et al. 2011](#_ENREF_21)). The remaining HIV-infected adults with unsuppressed viral load are still infectious and spreading virus in the community. Achieving high coverage of MC among HIV-negative males has also been challenging with most countries, including Botswana, unable to meet ambitious 80% coverage targets to date ([Curran, Njeuhmeli et al. 2011](#_ENREF_13)).

In summary, despite having the tools to end the HIV epidemic ([Fauci and Folkers 2012](#_ENREF_19)), insufficient scale, quality, and coordination of prevention interventions have significantly limited southern Africa’s ability to sufficiently curb the HIV epidemic. This has led public health experts to call for a “combination prevention” strategy that involves implementing multiple prevention interventions with known efficacy in a geographic area at a scale, quality, and intensity to impact the epidemic ([Merson, Padian et al. 2008](#_ENREF_51), [Piot, Bartos et al. 2008](#_ENREF_60)). Although most experts agree that synchronized implementation of a combination of prevention interventions is needed, there remains considerable debate about what components should be included in the essential combination prevention package ([Merson, Padian et al. 2008](#_ENREF_51), [Halperin 2009](#_ENREF_29)). Four evidence-based interventions (HTC, ART, MC and PMTCT) are recommended for scale-up by almost all prevention experts and will form the cornerstone of the combination prevention package proposed as part of this project (see Section 1.4 below).

- 1. **ART as a Component of the Combination Prevention Package**

Data show that higher levels of HIV-1 RNA (viral load) are independently associated with both higher rates of HIV transmission and more rapid HIV clinical disease progression and CD4 decline. This study therefore originally included the use of a modified “treatment as prevention” approach that targeted not only those adults who need ART for their own health, but also those adults who are most likely to perpetuate HIV transmission within the community (those with viral load ≥10,000 copies/ml). At the time of designing the BCPP in 2012 there was strong experimental evidence supporting initiation of ART at CD4≤350 cells/µL, or WHO Stage III/IV, but conclusive experimental evidence supporting patient benefit of ART initiation at higher CD4 counts, especially CD4 >500 cells/µL was lacking.

The 2013 WHO guidelines recommended initiation of ART in all HIV-infected adults with CD4≤500 cells/µL regardless of clinical stage. In an attempt to provide programmatic data for an anticipated change in CD4 threshold for ART initiation in Botswana and to enhance the power of the study to answer its primary objective, the threshold for ART initiation in the Combination Prevention arm was modified in early 2015 (from CD4≤350 to CD4≤500 regardless of viral load, in addition to offering expanded ART to persons with CD4>500 and viral load ≥10,000).

Shortly thereafter, in July 2015, the WHO announced that it would revise ART guidelines to recommend universal treatment (regardless of CD4 count or HIV disease severity). The WHO’s plans were informed by results from two clinical ART trials. One, the START trial, reported that early drug treatment of people with HIV cut their risk of serious illness or death by 57% ([Lundgren, Babiker et al. 2015](#_ENREF_85)). Another, the TEMPRANO study, released findings that early ART significantly decreased morbidity overall and when restricted to patients with baseline CD4>500 cells/µL ([Danel, Moh et al. 2015](#_ENREF_31)). WHO published an early release guideline on when to start ART and on pre-exposure prophylaxis for HIV in September 2015 ([World Health Organization 2015](#_ENREF_142)). The BCPP study team, with the support of the DSMB and funder, is revising the intervention treatment initiation criteria once more to take these new results and WHO guidelines into account. Universal Test and Treat (UTT) is therefore included in the combination prevention package with version 4.0 of the protocols.

In June 2016, Botswana treatment guidelines changed to UTT. All PLHIV living within the Enhanced Care communities (ECC) are now eligible to receive ART according to these updated Botswana national guidelines. Residents in the Combination Prevention communities will also continue to receive universal treatment as prevention.

Through additional modeling, the study team has adjusted effect size and power calculations to reflect a move to UTT in both the Combination Prevention and Enhanced Care arms, using a range of roll-out timelines. This revised modeling also uses estimates of coverage (by HTC, LTC, ART, MC) that are consistent with what has been observed in BCPP thus far, rather than using the higher coverage “targets”. Model-based estimates suggest that the impact of a change in treatment policy on the power to detect a difference in cumulative incidence (the primary objective of BCPP) may still yield at least 70% power (Protocol 1, v4.0, section 5.1.1), but the impact depends on the exact nature and timing of the implementation of changes in policy as well as other prevailing conditions.  The retention of this level of power even with Enhanced Care communities moving to expanded access to ART is at least in part attributable to the fact that BCPP is implementing multiple HIV prevention interventions (including higher HTC coverage, linkage to HIV care/treatment, etc.).

- 1. **Proposed Combination Prevention (CP) Package**

In version 4.0 of this protocol, the combination package includes universal treatment of all PLHIV along with a package of interventions that are designed to: 1) Identify all HIV-infected adults in CP communities, 2) reduce barriers to ART initiation, 3) support adherence and retention on ART, 4) increase capacity of clinics to support aggressive ART scale-up, and 5) enhance the use of both individual and clinic-level data to ensure maintenance of quality of HIV care and treatment.

The revised combination prevention package for this project includes:

- Rapid scale-up of HTC services, with a target of ensuring >90% of HIV-infected adults aged 16-64 have documentation of their HIV-infected status.
- Rapid scale-up of universal ART for all HIV-infected adults, with a target of ensuring that >93% of adults diagnosed with HIV infection are receiving ART.
- Rapid scale-up of retention in care and adherence interventions, with a target of ensuring that >95% of HIV-infected adults receiving ART are virally suppressed with HIV-1 RNA < 400 copies per ML.
- Scale-up of linkage to MC services, with a target of ensuring >60% of HIV-negative men (aged 16–49) are circumcised.
- Rapid strengthening of PMTCT services, with a target of ensuring >90% of women initiated on indefinite ART (Option B+) during pregnancy remain in care and on treatment post-delivery.

The targets for intervention communities in this version of the Protocol have been revised from the targets in Protocol 3, version 3.0. Targets for ART initiation and viral load suppression have been raised based on the higher than expected baseline levels of ART initiation and viral load suppression coverage as measured in BCPP BHS and HTC campaign. The MC target has been lowered from 80% to 60%. This change is in accordance with the DSMB’s 2013 recommendation based on concerns that the 80% target might not be realistic; results to date support these expectations.

To reach stated scale-up targets (cumulative over 3 years) several service uptake initiatives will be implemented, including (1) community mobilization to encourage HTC and MC uptake, (2) targeted HIV testing to include hard-to- reach populations, (3) strengthening linkage to care, treatment and/or PMTCT services for known HIV-infected adults not in care and newly diagnosed HIV-infected adults, (4) strengthening linkage to MC services for HIV-negative or HIV-unknown status males identified at HTC services, (5) strengthening retention in care and adherence to ART services at clinics (6) expansion and strengthening of HTC, ART, PMTCT services, and MC services through recruitment of additional health care personnel and counselors, and (7) strengthened monitoring and evaluation of prevention, care and treatment programs to ensure stated targets are reached.

Adults aged 16-64 will be targeted for service uptake because recent, nationally representative surveys of HIV prevalence and incidence ([BAIS 2008](#_ENREF_5)) suggest that HIV incidence remains high (>1%) throughout this age group. Modeling exercises suggest that meeting scale-up targets by the end of two years of program implementation, and maintaining treatment coverage thereafter, could result in community HIV incidence reductions of 40% by the end of three years of implementation.

- 1. **Relationship Between Intervention Study Design and Impact Evaluation**

A separate protocol (BCPP Evaluation, Protocol #1) describes in detail the proposed plans for evaluating the impact of the combination prevention (CP) package on adult population level HIV incidence. In brief, a randomized design was proposed to provide experimental evidence of CP package impact and because population-level HIV incidence is the outcome of interest, a community (rather than individual) randomized trial is proposed. Thirty study communities have been selected and matched into pairs. Pair-matching is done to improve balance between study arms and statistical efficiency ([Hayes and Moulton 2009](#_ENREF_32)). Communities within pairs were randomized to Combination Prevention Community and Enhanced Care Community (CPC and ECC) arms. Following community matching, a baseline 20% household survey (BHS) was implemented to recruit an HIV Incidence Cohort (HIC) of eligible, consenting, and HIV-negative adults aged 16-64. The HIC will be followed annually during Annual Household Survey (AHS) visits to assess cumulative HIV incidence over 30 months. Study participants who are not eligible for the HIC will also be followed annually in the AHS, to assess, in both study arms, uptake of HTC, ART, MC services and PMTCT services in each arm and to describe major health outcomes (such as death). Cumulative incidence will be compared between study arms to assess CP package impact.

Combination prevention package coverage in the community will be estimated at baseline (in the 20% BHS) and through a large end of study survey (ESS). In addition, during study conduct, progress in reaching target coverage levels will be captured from monitoring data collected as part of routine programmatic activities.

**2.0 STUDY OBJECTIVES**

**Primary Objective**

1. To implement the combination prevention (CP) intervention package in CPCs and describe uptake of these interventions (expanded HTC, strengthened MC, expanded HIV Care and Treatment, and strengthened PMTCT services).

**Secondary Objectives**

1. To describe retention in the clinical cascade of care from HIV diagnosis to viral suppression for HIV-infected persons.
2. To describe uptake of expanded ART and clinical outcomes including viral suppression among HIV-positive persons receiving ART (UTT).
3. To examine demographic, clinical, and social/behavioral facilitators and barriers to uptake of CP interventions.
4. To examine demographic factors, including community of residence, that affect access to health care services.
5. **OVERVIEW OF STUDY METHODS**

As described in the introduction, the over-arching design is a pair-matched community-randomized trial (CRT). The full details of the CRT, including how communities were selected, how they were matched, community randomization, the BHS, enrollment of the HIV incidence cohorts (HIC), and the end of study survey (ESS), are described in the BCPP Evaluation Protocol. This protocol describes the interventions implemented in 15 communities randomly selected from the 30 study communities *(****Appendix C2),*** monitoring and evaluation (M&E) plans, and the program data systems.

1. **STUDY POPULATIONS**

**4.1 Selection of Study Communities**

Thirty communities (***Appendix C2***) in three geographic areas in Botswana: (1) around Gaborone in the south east, (2) around Francistown in the north east, and (3) in the region around Mahalapye, Serowe, and Palapye in the central eastern region, are included in the study. These communities were selected based on two criteria: (1) communities needed to be of sufficient size to meet sample size requirements for the HIV Incidence Cohort (described in BCPP Evaluation Protocol), and (2) implementation of the proposed combination prevention package needed to be feasible. Feasibility requirements are listed below:

- **Community Criteria**

All communities must:

- Have a health center from which HIV care and treatment, PMTCT, or HTC can be provided.
- Be reachable by road via a <8 hour drive from Gaborone or Francistown. (CDC and the Ministry of Health have permanent, well-staffed offices in Gaborone and Francistown, while the Harvard Chan School has a permanent office in Gaborone. This distance parameter makes the study easier to implement and monitor for quality control purposes).
- Have a clear geographic boundary such that it is reasonably easy to identify a household as either being part of, or outside of, the study community.

These inclusion criteria resulted in the exclusion of urban areas from the study. Approximately 61% of Batswana (plural noun for Botswana citizens) live in urban areas with a rate of urbanization of about 2.3% per year (annual rate of migration from rural or peri-urban regions into urban areas). Restricting the study to rural and peri-urban areas, besides being necessary for study feasibility, should not limit the generalizability of study findings to urban areas. If the combination prevention package is shown to be effective at reducing adult HIV incidence, the principle of scaling up combination prevention package coverage for prevention effectiveness should be applicable to urban settings.

The 30 study communities selected meet the criteria described above. The average total population size of each of the targeted communities is 5,855. Assuming an average community size of 5,855, we estimate a total population in all 30 study communities of about 175,664 people; 87,832 persons in the CPC arm of which ~58% of community residents are aged 16-64 ***(Appendix C2/C3).*** HIV prevalence among residents aged 16-64 is estimated at 25%, resulting in an estimated 25,471 HIV-infected residents aged 16-64 (12,736 per study arm).

- 1. **Matching of Study Communities**

Due to the small number of communities that can be enrolled in a community randomized trial for cost and feasibility reasons, the likelihood of imbalance of potential confounders between study arms is increased. A restricted randomization, matching, and stratification were considered as strategies to ensure balance between arms. A pair-matched design was chosen because:

- - This approach is more likely than stratification to achieve balance between study arms;
  - Matching has the added advantage of improving statistical efficiency compared with restricted randomization, and;
  - Chosen matching covariates (see below) are quite closely correlated with the outcome of interest (adult HIV incidence), making a matched design of similar or increased statistical efficiency compared with a stratified design, even though a matched design entails greater loss of degrees of freedom.

Possible disadvantages of a pair-matched design include:

- - Loss of both communities in a pair if one community drops out.
  - It is not possible to assess whether the intervention effect varies between matched pairs, whereas, in a stratified design, opportunity to assess effect modification across strata would be possible.

The following matching criteria were chosen:

- **Community size**: Although community size in Botswana may not be associated with the outcome of interest (HIV incidence), achieving sample size balance between arms improves statistical efficiency.
- **Baseline Access to Health Services including ART**: Access to ART is associated with HIV incidence ([Montaner, Lima et al. 2010](#_ENREF_54), [Tanser, Bärnighausen et al. 2012](#_ENREF_66)). Although some communities within matched pairs have access to primary care clinics and others have access to primary care hospitals, in Botswana this does not appear to represent a significantly different level of access to health services.
- **Age structure**: Adult HIV prevalence and incidence are correlated with adult age ([Central Statistics Office 2009](#_ENREF_9)). Age structures are very similar within the study community pairs. If we use the proportion of adults (16-64) who are in the highest risk for HIV infection (25-34) as an indicator of age structure, the median difference in this proportion is 2% (range 0-7%). *[Note: we do not have age structure data for Gumare, Shakawe, Mandunyane, or Mmadinare, but based on the age structure of the other 26 selected communities, do not expect any significant differences in age structure within the matched pairs].*
- **Geographic location** as measured by proximity to major urban centers ([Hayes, Mosha et al. 1995](#_ENREF_31)). Geographic locations of communities within pairs, as estimated by average distance to the nearest large towns, are quite similar. For example, across 30 communities, the range of average distances to nearby urban centers ranges from 18-380 kilometers. However, following matching, the median difference in distance to an urban center between communities in a matched pair is 20 kilometers (range 3-150).

Access to community-specific measures of HIV prevalence [e.g., HIV prevalence measured from antenatal care (ANC) clinics] were not available prior to community selection/randomization. Previous successful pair-matched CRTs ([Hayes, Mosha et al. 1995](#_ENREF_31)) where HIV incidence was the outcome of interest have also addressed the problem of absent baseline HIV prevalence estimates by using geographic location and other proxies of HIV prevalence as matching criteria.

- 1. **Eligibility of Community Members** The following definitions of the study community will be used in the protocol:
- *Botswana Citizen:* A Botswana citizen is anyone who can produce proof of Botswana citizenship. (Usually this is the Omang number or Omang application receipt).  The Omang number is needed in Botswana to access routine government services.   It is anticipated, based on a community survey in Mochudi, and the 2011 census ([Census 2011](#_ENREF_20)), that less than 3% of residents will be non-citizens.

- *Spouse of Botswana citizen*: A spouse of a Botswana citizen is a person who can produce evidence of being married to a Botswana citizen, usually a marriage certificate, and is therefore eligible to receive free medical services in Botswana.
- *Permanent study community resident:* An individual who reports spending on average >14 nights/month each month in that community for the past 12 months or an individual who recently moved into the community and reports spending on average ≥14 nights each month since arriving.

- *Part-time study community resident:* An individual who reports spending 3—13 nights (on average) each month in that community for the past 12 months or an individual who recently moved into the community and reports spending on average 3-13 nights each month since arriving.
- *Non-residents:* An individual who reports living outside of the community but is receiving treatment in the CP community clinic as well as those individuals with an unknown community of residence who are receiving treatment in the CP community clinic.

- 1. **Enhancement of services in *all* study communities**

One of the goals of BCPP is to provide enhancement of HIV prevention, care and treatment services in non-intervention (ECC) communities as well as expansion and strengthening of these services in the combination prevention communities. This goal is primarily motivated by two factors:

1. A commitment to offering the enhanced care communities tangible benefits from participating in this community randomized trial, and
2. The imperative to bring the standard of practice in all study community clinics close to the standard of care, so that the BCPP study is assessing the effect of the combination prevention interventions on HIV incidence.

For the purposes of BCPP, the term ‘enhanced’ will be used to signify activities done in all study communities (including Enhanced Care Communities) for the purposes delineated above, whereas the terms ‘strengthened’ and ‘expanded’ will be used to signify activities done in Combination Prevention Communities only, as part of the study’s combination prevention intervention package. ‘Strengthened’ will signify that activities are already part of national policy and practice, whereas ‘expanded’ will signify that activities are beyond current national policy and practice (for example, ‘expanded’ antiretroviral therapy in the CPCs).

The following enhancements will be provided in all 30 study communities, including the ECCs:

1. Baseline and Annual Household Surveys (Evaluation Protocol): As part of the BHS that occurs in all 30 communities (in both study arms), ~20% of adults are offered home-based HIV testing, point-of-care CD4 count if treatment-naïve, viral load testing, and referral for HIV care and treatment, PMTCT services, MC, and other health services as appropriate (TB screening, for example). HIV-infected persons who were ART naïve at entry to BHS or who seroconvert between study visits and are ART-naive will also have annual point of care CD4 and viral load tests, and these results will be shared with participants/clinics respectively.  HTC is also offered to household residents who do not wish to take part in the BHS/AHS, either by the BHS study team, the HTC campaign team, or by referral to local clinics where HIV testing is available.
2. Clinical care quality assessment and quality improvement: BCPP and/or CDC Botswana will work through the Ministry of Health to support clinical quality care assessment and improvement. Existing or new Ministry of Health and CDC tools and quality improvement documents will be used or expanded in this effort.
3. Clinical data system support: BCPP will provide technical assistance to the Ministry of Health to support improved implementation and utilization of clinical data systems (primarily PIMS2 and IPMS) in all 30 study communities.
4. Laboratory support: BCPP will provide technical assistance to Ministry of Health to help laboratories supporting clinics in all 30 study communities to perform at a level sufficient to meet the standard of care. Existing or new Ministry of Health and CDC tools and quality improvement documents may be used or expanded in this effort.
   1. **Eligibility Criteria for Inclusion in Interventions in CP Communities**

This protocol provides the inclusion and exclusion criteria for access to the services provided as part of the Combination Prevention intervention package. Adults aged 16-64 will be targeted for service uptake because recent, nationally representative surveys of HIV prevalence and incidence ([BAIS 2008](#_ENREF_5)) suggest that HIV incidence remains high (>1%) throughout this age group.

For clinical services, only those who are Botswana citizens or spouses of Botswana citizens can receive free services from Ministry of Health facilities. Non-citizen/non-spouse of citizen HIV-infected adults identified during testing will receive a referral to the health clinic in their community of residence, but will not be eligible for free services from Ministry of Health facilities, and thus will not be eligible for strengthened free services in intervention clinics.

Investigators propose that eligibility for interventions in the CPC arm be grouped into three categories (a) eligibility for expanded HTC services and (b) eligibility for strengthened linkage-to-MC services, and (c) eligibility for expanded treatment and follow-up.

1. *Eligibility for Expanded HTC*

All adults >16 who wish to receive HTC services at health facilities, mobile testing units, or door-to-door programs in combination prevention communities (CPCs), are eligible to receive these HTC services. The rationale for proposing that non-residents of the CPC be able to access expanded HTC services is to improve community acceptance of the door-to-door and mobile HTC activities. Preliminary discussions with community leaders suggest that restricting access to a service like HTC which is being delivered to family homes and neighborhoods would be met with considerable resistance and community discontent.

Non-citizens of Botswana will also be provided HTC services in the CPC. Non-citizen/non-spouse of citizen HIV-infected adults identified during testing will receive a referral to the health clinic in their community of residence to connect them with available fee-based services including treatment.

1. *Eligibility for Strengthened Linkage-to-MC services*

Noncitizens will continue to be eligible for enhanced linkage- to-MC services if they meet other eligibility criteria. Under the national MC programs, noncitizens who present directly to the MC tent requesting MC will be offered free services. Thus, all adult men 16 years or older who are HIV-negative or have unknown HIV status are eligible for strengthened linkage-to-MC services including men who decline HIV testing or have indeterminate results. Under current national guidelines, boys 13-15 years old will not be actively mobilized or included in linkage-to-MC though they can continue to receive free MC services at national locations if they present with parental permission.

1. *Eligibility for Universal Treatment*

Universal treatment will be offered to all HIV-infected adults aged 16-64 who are Botswana citizens or spouses of Botswana citizens. HIV-infected persons referred through the study via BHS/AHS, HTC or routine HIV testing (RHT) as well as existing patients already enrolled in care at the CPC clinics will be offered expanded treatment. The Infectious Disease Care Clinics (IDCC) treat all Botswana citizens and spouses of Botswana citizens regardless of the community in which they reside. Therefore, both residents and non-residents of the community will be included.

- 1. **Rationale for Exclusion from Interventions in CP Communities**
- *Rationale Surrounding Need for Botswana Citizenship or for Being Spouse of Botswana Citizen:* Although the Botswana Government provides free HTC services to non-citizens, free access to health services, including ART and PMTCT is restricted to a) persons who can produce valid documentation of Botswana citizenship or b) spouses of Botswana citizens. Combination prevention interventions will therefore not be available to non-citizens of Botswana unless married to a citizen, except in the case of HTC and MC services. The exception for MC will be made to bring study goals closer to aligning with WHO goals for protection of communities through MC provided to 60% of HIV-uninfected men - regardless of citizenship status - as noncitizens living in the community may share the same sexual networks with citizens.

Additionally, men who decline HIV testing may actually be those at greatest risk for HIV acquisition and thus could benefit most from MC. If such men were not offered MC and were later found to be positive at end of study they would have represented a missed opportunity for prevention.

- *Rationale for Age Limits***:** Persons <16 years of age will be excluded from study participation because our intervention is targeting adults, and relevant study outcomes will be measured among adults. In Botswana, adults are considered those aged 16 or older. For example, all persons aged >16 can access medical services, including HTC, without guardian consent. Although individuals aged 16-17 can access medical services without guardian permission, participation in research still requires guardian permission, because the age of consent for research in Botswana is 18. Therefore, eligible 16-17 year-olds will be asked for assent to participate in research activities **only after** permission has been provided by the guardian.

1. **SAMPLE SIZE AND SAMPLE SELECTION METHODS**

The primary objective for the intervention protocol is to implement and describe uptake of CP interventions in CP communities. Combined (BHS and HTC) data for 7 intervention communities was used to derive average denominator sizes for the estimates of interest. These community-average sample sizes were multiplied by 15 to yield an estimate of the total denominator (see Table 1). For the estimate of uptake of HTC, we used the number of participants interviewed to be conservative, although estimates of HTC coverage will be extrapolated to persons not interviewed.

Table 1 presents confidence limits for all of the indicators of uptake of CP interventions for protocol 3, using targets presented earlier in the protocol. Sample size calculations were done using a simple binomial method to obtain standard errors and then multiplying standard errors by the square root of the variance inflation factor (VIF), which is [1+(m-1)*ICC], where m is the average number of members of each group (here, participants within community), and ICC is the intraclass correlation coefficient, reflecting the magnitude of the correlation within communities for the outcome measure of interest. No published ICCs were available for these outcomes, so we used one small and one large value, based on prior publications reporting ICCs for behavioral variables in HIV and STD studies (Zhang et al., 2013 and Pals et al., 2003).

**Table 1. Precision for 95% confidence intervals for uptake of CP interventions in CP communities**

| **Outcome** | **Denominator**  **(per community)** | **Denominator (Total across CP communities)** | **ICC** | **Coverage** | **95% Confidence Interval** | **CI Width** |
| --- | --- | --- | --- | --- | --- | --- |
| HTC | 2469 | 37035 | .02 | 90% | 87.8, 92.2 | 2.2 |
|  | 2469 | 37035 | .05 | 90% | 86.5, 93.5 | 3.5 |
| PMTCT | 50 | 750 | .02 | 90% | 87.0, 93.0 | 3.0 |
|  | 50 | 750 | .05 | 90% | 86.0, 94.0 | 4.0 |
| MC | 794 | 11910 | .02 | 60% | 56.4, 63.6 | 3.6 |
|  | 794 | 11910 | .05 | 60% | 54.4, 65.6 | 5.6 |
| ART | 528 | 7920 | .02 | 93% | 91.1, 94.9 | 1.9 |
|  | 528 | 7920 | .05 | 93% | 90.0, 96.0 | 3.0 |

Table 1 shows that where sample sizes are large and we expect rates of uptake closer to 100% (HTC and ART), precision is excellent. Where sample sizes are small (PMTCT) or rates expected closer to 50% (MC), we have less precision for estimates, but confidence interval widths are still less than ±6%. As the rate of the outcome variable of interest moves away from 50%, standard errors and confidence interval width will decrease. With a larger ICC, the variance inflation factor increases and confidence intervals will widen.

**6.0 INTERVENTION PROCEDURES**

**6.1** **Overview of Intervention Strategy**

Interventions described below are intended to: 1) be consistent with new WHO guidelines on treatment of HIV infection (World Health Organization, 2015), 2) streamline clinic procedures to accommodate expansion of treatment to all PLHIV, and 3) incorporate lessons learned during the first year of BCPP. Targeted strategies will be implemented to meet UNAIDS goals of 90% of PLHIV know their status, 90% of PLHIV who know their status are on treatment, and 90% of PLHIV on treatment are virally suppressed. Through reaching the UNAIDS goals, it is anticipated that there will be a reduction in HIV incidence in the CP communities as assessed in Protocol 1. The proposed BCPP interventions will demonstrate UTT and introduce a streamlined approach to finding, testing, and starting PLHIV on immediate treatment and retaining them on effective treatment over time. Specific engagement activities as well as the specific interventions being deployed are outlined in detail below.

**6.2 Stakeholder Involvement, Community Engagement, and Community Mobilization**

To enhance communities’ acceptance and effectiveness of the combination prevention package, local government and community stakeholders will be highly engaged. Program implementers, including MOH at central-, district-, and facility-level, and NGOs supporting the MOH’s public health mission in study districts, have been engaged in the planning and continue to be involved in implementing all combination prevention interventions. These governing bodies include:

- *National Government:* MOH, CDC and Harvard Chan School investigators and program planners have presented project plans to (1) the Parliamentary Sub-committee on Health, (2) the Parliamentary Sub-committee on HIV/AIDS, and, (3) the House of Chiefs (a body of traditional leadership made of representatives of all communities in the country).
- *District Government:* Study communities are distributed across 13 of 29 districts in Botswana. Meetings and consultations have been held with the District AIDS Coordinator, District Multi-Sectoral AIDS Committee, and the District Health Management Teams.
- *Community Government:* At the community level, village political leadership has been consulted, including the traditional leadership of *Dikgosi* and *Dikgosana* (Chiefs and sub-chiefs), the Village Development Committees (VDCs), church leaders, and sporting clubs. Project plans were shared and discussed prior to initial implementation and ongoing support for the project is sought.

Investigators meet regularly with members of study Community Advisory Boards (CAB) to discuss community perceptions of the project. Minutes of these meeting are documented and shared with the study principals. Specific issues raised during CAB meetings are addressed as needed. There are four CABs located in the following regions: *South*, *Central, North, and Northwest.* The CABS have representatives from the study communities in that region.

Community mobilization activities are conducted in all communities; however, the scope and duration of the activities are greater in the CPCs. Activities focus primarily on increasing uptake of HTC and MC so that HIV-infected adults can access treatment and/or PMTCT and HIV-negative adults can access MC. Some of the methods to improve uptake of services include:

- - - Public announcements from vehicles with loudspeakers (very common in Botswana),
    - Community (kgotla) meetings, civic society activities, schools, and clinics as a platform to inform the community.
    - Door-to-door canvassing of the community informing community members of the services that are available in the community, location and duration.
    - Specific mobile activities (e.g., location of an HTC caravan near work and sporting locations or in residential areas after business hours (e.g., after 5 pm) targeting hard to reach populations like men.

**6.3** **HIV Testing and Counseling (HTC)**

1. **Target**: 90% of PLHIV know their status.

1. **Strategy:** Based on the first round of HTC in BCPP, the HTC strategy is being modified for years 2 and 3. During the first year of HIV testing, approximately 70-% of HIV+ persons in the community were identified through concentrated testing efforts lasting 6-8 weeks. During the 2^nd^ year of the study, HTC activities will be provided through continuous availability of services in the community and will focus on 1) finding incident cases of HIV infection by retesting persons found to be negative during the 1^st^ year; 2) finding the 25-30% of community members missed by the 1^st^ year of testing in BCPP; and 3) identifying HIV+ persons who are new arrivals to the community.

The activities to fulfill the first objective of finding incident HIV cases include 1) repeating door-to-door HIV testing during year 2 in all homesteads from year 1; 2) monthly mobile testing at targeted community locations; and 3) strengthening routine HIV testing (RHT) in health facilities with a focus on antenatal clinics, outpatient departments and IDCC. To fulfill the 2^nd^ objective, targeted testing activities will be used to find community members missed in the first year of testing in BCPP. Based on data collected in year 1 of BCPP, approximately 50% of men and 30% of women are missed through home-based testing. Targeted testing strategies to locate those missing in year 1 will include workplace testing, home-based testing on holidays and weekends, mobile testing at venues frequented by men and large-scale community activities that attract the majority of community members. To fulfill the 3^rd^ objective, new arrivals to the community who are HIV+ will be found through re-enumeration of households and HIV testing while going door-to-door. New arrivals will also be identified during community mobile testing and large-scale community events that offer HIV testing. The HTC strategy for year 3 of BCPP will be based on findings from year 2 of the study, but is likely to focus on targeting populations missed in year 2.

In order to reach the target of the 90% of PLHIV knowing their status, cost-effective community-based HIV testing strategies are needed to find unidentified HIV-positive persons who are not accessing health care facilities, such as young women and men. An understanding of the incremental costs between strategies is key to determine the optimal mix of strategies to implement. Therefore, a cost analysis of different HTC models implemented through BCPP will be undertaken.

1. **Specific HIV Testing and Counseling Services:**
2. ***Home-Based HTC*:**  HTC counselors will go door-to-door and request verbal consent to: (1) map household GPS location, (2) record numbers of persons in each household, their age, and sex by interviewing the head of household or, if the head of household is absent, a household member 18 years of age or older (***Household Enumeration Form***), and (3) offer HTC services **(*HTC Intake Form*).** The following individuals will **not** be offered HTC:
   1. Adults who have documentation of an HIV-positive status
   2. Adults who have documentation of HIV-negative results obtained within the last 3 months of the HTC encounter. (Documentation of HIV test results are recorded on the ***HTC Intake Form***)
   3. Persons <16 years old

If a household member is absent, the team conducting the home-based HTC activities will ask about an appropriate time to return to the household (***Household Enumeration Form***). Up to 3 repeat visits will be scheduled.

1. ***Mobile HTC*:**  Mobile HTC caravans and tents will be placed in prominent locations of the community [e.g., at kgotlas (community meeting locations), or shopping centers]. Community mobilizers will engage the community via door-to-door ‘mobilization’ visits and in high traffic areas in the community such as shopping and transport areas and invite residents to get tested in the mobile units and tents. Lay counselors and support staff will be stationed in mobile venues consisting of a waiting area, private HTC area and a phlebotomy area. HTC will be offered to all individuals (residents and non-residents, citizens and non-citizens) who are not known to be HIV-infected according to national guidelines (i.e., adults who do not have an HIV test result card indicating HIV-positive status). Persons who tested will be asked to keep the card with their results safely for any future contact, e.g., during subsequent household visits (below).
2. ***Routine Facility-Based HTC*:** Routine HTC activities at health facilities will be strengthened by training facility-based counselors to offer RHT as part of integrated routine services in outpatient, IDCC, and antenatal (ANC) clinics. Facility-based counselors will be encouraged to provide testing to all adults receiving health care services at the clinic and to offer testing to all friends, family members, relatives, visitors, etc. (so-called non-patients) accompanying the patient to the clinic. In addition, the study will assist MOH ANC staff with strengthening of 3^rd^ trimester re-testing among pregnant woman (see section 6.6). Routine HIV testing data will be extracted from PIMS2/IPMS to monitor coverage of testing in health facilities.
3. **Targeted Testing:** HTC for groups that are difficult to access through home-based, clinic-based, or other routine services will be targeted by providing services where these groups work or congregate and by offering HTC services at large, well-attended community events such as barbecues, health fairs, concerts, etc.
4. **Additional Services for Persons Testing HIV-Positive:** All HIV positive adults not on treatment are eligible for ART under current national guidelines so will not need to consent for treatment in the UTT intervention. HTC counselors are trained to integrate treatment preparation, adherence and readiness messages into their post-test counseling for HIV-positive persons to begin preparing participants for initiating ART at their first clinic visit. In addition, they will be counseled to expect a confirmation test for HIV positive results before initiating ART which will be conducted at the IDCC. All participants of HTC will be tested using the MOH rapid testing algorithm along with pre-test and post-test counseling that is recommended in the national guidelines.
5. **Additional Services for Persons Testing HIV-Negative**: The following services will be offered to newly identified or known HIV-negative adults:

- Screening of all adults for TB, with a simple screening question, and referral of TB suspects to the nearest clinic for TB testing. TB suspects will be prioritized for evaluation at the clinic. In Zimbabwe and Zambia, this has been shown to reduce community prevalence of infectious TB and was well-accepted by the community ([Corbett, Bandason et al. 2010](#_ENREF_11)) .
- Encourage MC uptake by HIV-negative males through linkage-to-MC services. These services also are offered to men with unknown HIV status. Further details of the linkage-to-MC service are provided in Section 6.5 below.
- HIV-negative pregnant women will be encouraged to enroll in ANC care and be retested in their 3^rd^ trimester.

**6.4 Linkage to HIV Care and Treatment**

**a) Targets:**  80% of adults identified as newly diagnosed or not currently in care are registered at the local study IDCC or are documented in care at another IDCC within 90 days of BCPP interview and 90% within 180 days of BCPP interview

**b) Strategy:** To ensure that 90% of persons identified as HIV-infected initiate ART, persons newly identified as HIV-infected or PLHIV not connected to care and treatment must be linked to an IDCC. Data from the first year of BCPP suggest that about half of PLHIV link to care with very little prompting or follow-up. Another 50%, however, require additional supports. The linkage intervention package for BCPP includes: 1) counseling after diagnosis on the importance of immediate treatment and appointments made for PLHIV not already in care; 2) appointment reminders; and 3) tracking and tracing of persons who miss appointments at the IDCC. In addition, to enhance linkage rates, patients wishing to receive care/ART outside of their community will be able to link to any MOH IDCC.

**c) Specific Activities:**

1. **Counseling After Diagnosis and Scheduling Appointments at IDCC:** Counselors will be available to: (1) provide immediate psychosocial support to help clients cope with their HIV-positive test result, (2) counsel participants on the importance of early entry into HIV care or PMTCT services and prepare them for treatment initiation and adherence at first clinic visit, and (3) help clients establish and achieve plans to enter care by identifying and troubleshooting real and perceived barriers to care ([Gardner, Metsch et al. 2005](#_ENREF_24), [Gardner, Marks et al. 2007](#_ENREF_23), [Craw, Gardner et al. 2008](#_ENREF_12), [Gardner, Marks et al. 2009](#_ENREF_22)). Appointments at IDCCs in intervention communities will be scheduled as soon as possible for newly diagnosed individuals or PLHIV who are not enrolled in care and treatment. Some PLHIV may prefer to obtain care and treatment services at a clinic outside their community for privacy, proximity to job, or other reasons. For those PLHIV who choose to obtain care outside the study community, verification of enrollment in care will be obtained from the MOH data systems.
2. **Appointment Reminders:** PLHIV will be sent short message service (SMS) reminders about their upcoming appointments.
3. **Tracking and Tracing:** A record of all patients referred to care by BHS, HTC and RHT will be submitted to Site Coordinator at the local IDCC. The Site Coordinator at the IDCC will access names of those referred and will track them to ensure they keep their first appointment. Names and contact information of those who don’t keep their appointments will be turned over to the community counselor for tracing and case management. The intensified participant tracing program includes telephone calls and home visits for persons who missed a scheduled appointment up to 180 days after either diagnosis or BCPP interview for known HIV positive persons (***Participant Tracing Log***).
4. **Counseling, Appointments and Tracing for PLHIV Identified who are already on ART**

- Patients who are on ART, enrolled in care at the study IDCC, are not late for an appointment, and have a future appointment scheduled will be encouraged to remain adherent to ART and appointments.
- Patients who have initiated ART and are enrolled in care at the IDCC and are late for their next appointment or who do not have a future appointment scheduled will be (1) counseled on the importance of adherence and appointment keeping, (2) given a new appointment at the clinic, and (3) have phone number and residential address information obtained to facilitate follow-up (retention tracing of participants already in care and late for their scheduled appointments is described in section 6.7 below).
- Patients who are on ART and not enrolled in care at the study clinic will be counseled to remain adherent to ART and in care; they will also be informed of expanded and strengthened services available at the study clinic and will be given an appointment to the study clinic. Follow-up for these patients will be limited to only an SMS reminder for their appointment. Verification of the patients’ enrollment at another clinic outside the study community and ART status will be obtained from MOH data systems.

1. **Retrospective Tracking and Tracing of PLHIV who Did Not Link to Care:** Given the change in the 2016 Botswana national treatment guidelines, HIV-positive participants not on ART who did not link to care from Round 1 (October 2013 – February 2016) will be retrospectively contacted by the Ministry of Health clinic staff or a community counselor and informed that all HIV-positive persons are now eligible for ART, regardless of CD4 count or condition. Participants from Round 1 and Round 2 (June 2016 – present) who previously did not give permission for follow-up may also be contacted, as the new guidelines recommend tracking all HIV-posiitive persons identified to care and treatment sites. Specifically,
   - Contact with the participant will be attempted by a Ministry of Health clinic staff member or a community counselor to inform the participant that the national guidelines have changed and all persons that are HIV-positive are now eligible for ART. They will also be informed that it is critical for their health and the health of their partners and children to get on ART as early as possible, and that the clinic offers services to help community members get into care and on treatment. Staff will use the updated talking points from the Linkage to Care SOP – revised (see Appendix XX, Section 3.8).
   - Participants will be given an appointment at the clinic with a health care provider and will be encouraged to keep it.
   - If there is no current phone number for the participant, a home visit will be conducted by a clinic staff member or a community counselor. Up to 3 attempts to contact the participant will be made through alternating phone and home visits.
   - Participants from Round 1 who have not linked to care will be contacted beyond the follow-up period specified in the protocol (e.g., 180 days) in order to ensure the new guidelines are being implemented with all participants. Paricipants from Round 2 who were enrolled during the time between the introduction of the new guidelines and field implementation (November 2016) may also be followed beyond the 180 day window.

A note to file will be documented at each of the 15 intervention sites noting the procedural changes and follow-up provided to those individuals who previously did not link and declined follow-up.

**6.5 Male Circumcision**

**a) Target:**  60% of HIV-negative 16-49-year-old males are circumcised

**b) Strategy:** BCPP’s SMC intervention in the second year will be streamlined from the full-service campaign model used in the first year to a program that mobilizes and links interested men to existing services in nearby locations. MC services are expected to be provided in five of the 15 CPC sites using PEPFAR programmatic support. In all other districts, nearby services funded by Global Fund or MOH will be used, though distances to these services from CPCs is likely to vary. All CPCs are expected to have at least one service option available. Therefore, BCPP funding will not be used to support direct service delivery but to mobilize and link men in BCPP communities to existing MC services. Modified SMC services are expected to begin when the second round of BCPP interventions are implemented, approximately February 2016.

**c)** **Specific Activities:**

**1) Mobilization:** In the first round of BCPP activities, SMC depended heavily on community-based mobilizers and referrals from the BCPP HTC Campaign and BHS to generate client demand. A basic level of this ongoing mobilization will continue. HTC and BHS will refer men to community mobilizers by passing contact information with consent; the mobilizers will then follow up with these men and link them to nearby services. Mobilizers will also work directly with communities to mobilize men for services. Incentives for mobilizers through a performance-based contract may be utilized to improve mobilization efforts. In addition to encouraging potential clients to obtain MC, mobilizers will disseminate service information about linkage sites, transport schedules, and assist with coordinating transportation.

**2) Coordination:** A small number of full-time coordinators will oversee all the CPCs. Their responsibilities will include identifying and liaising with locally-available SMC services to confirm their schedules and notify them of expected clientele numbers on transportation days; supervising mobilizers; coordinating transportation; and follow-up with service sites to collect the necessary data. A senior BCPP staff member will keep the MOH SMC coordinator up to date on these activities.

**3) Transportation:** In each CPC, transportation will be offered at least monthly to nearby SMC services, on days of the week determined to be optimal. Transportation will also be provided for day-2 and day-7 follow-up visits, and will remain available for adverse event emergencies.

**4) Data collection:** Individual-level data will not be collected at SMC service provision sites by BCPP, but aggregate data will be requested from these sites. Specifically, at these sites, the coordinator will make attempts at least quarterly via physical visits and register reviews to ascertain how many HIV-negative/unknown men 16-49 years of age from the CPC were served. The coordinator will also collect aggregate data from the transfer logs to gather numbers of men transported to SMC services. Finally, the coordinator will collect aggregate data on number of men referred and mobilized from demand creation records completed by HTS and BHS staff as well as SMC mobilizers (***Male Circumcision Tracking Sheet***).

**6.6 Identification, Linkage, and Treatment of HIV+ Pregnant Women**

**a) Target:**  90% of women initiated on indefinite ART (Option B+) during pregnancy remain in care and on treatment post-delivery.

**b) Strategy:** Strengthening PMTCT activities is important to ensure that HIV-infected pregnant women are identified, linked to care, and remain on ART post-delivery. Currently, Option B+ is the national standard of prevention of mother-to-child transmission in Botswana. Therefore, BCPP intervention and control communities will provide Option B+ interventions for all HIV+ pregnant women. The BCPP program for pregnant women will focus on strengthening three activities in PMTCT settings in intervention communities: 1) strengthening of third trimester retesting within the ANC; 2) linkage to HIV treatment for all HIV-infected pregnant and post-partum women; and 3) retention on ART post-delivery.

**c) Specific Activities:**

**1) Strengthening third trimester retesting:** BCPP staff work with MOH clinic staff to strengthen capacity to identify women who are due for third trimester retesting. MOH clinic staff will use client contact information from the ANC register to make phone calls and schedule appointments with all HIV-negative pregnant women who have not completed third trimester retesting. MOH clinic staff will keep a record of clients called for third trimester retesting. Third trimester retesting will be monitored through PIMS/IPMS by the IDCC clinic.

**2) Linkage and Retention for HIV-infected Women:**  Ensuring HIV-positive pregnant women receive ART and remain on ART following delivery is a key priority. All HIV positive pregnant women identified through the BHS and HTC campaigns are referred to the IDCC for HIV care and treatment. In addition, HIV+ post-partum women are referred to the IDCC to continue ART indefinitely (Option B+). Those who do not keep IDCC appointments will be tracked by the IDCC Site Coordinator and followed by community counselors to ensure they link or re-link to the IDCC post-delivery. Similarly, retention activities will track women who have begun Option B+ to ensure that they stay in care and on ART indefinitely. Those who are 14 or more days late for an appointment or pharmacy pick up will be contacted by MOH clinic staff and/or the IDCC Site Coordinator (***Linkage, Retention and Adherence Tracing Verbal Consent Script***) and community counselors will provide supportive counseling and new appointments through phone and home visits (***Participant Tracing Log)*** as outlined in section 6.4.

**6.7 HIV Care and Treatment**

**a) Targets:**

1) 93% of PLHIV aged 16–64 who know their status are receiving ART

2) 95% of PLHIV aged 16-64 who are on ART are virally suppressed

**b) Strategy:** Prior versions of BCPP Protocol 3 introduced anti-retroviral treatment outside Botswana national guidelines (CD4 < 350, WHO stage III or IV). With the revised WHO recommendations to treat all HIV-infected persons, Version 4.0 of Protocol 3 proposes to start all HIV+ adults in CPCs on immediate treatment and retain them on treatment (UTT). To demonstrate methods for implementation of UTT for later adoption by the Botswana MOH, BCPP will embed UTT treatment interventions within the national ART program (MASA). The package of interventions described below are designed to 1) reduce barriers to ART initiation by simplifying procedures and making services easily available 2) increase capacity of IDCCs to support aggressive ART scale-up, and 3) enhance the use of both individual and clinic-level data to monitor uptake of HIV care and treatment.

**c) Specific HIV Care and Treatment Activities:**

**1) Confirmation of HIV status:** All HIV+ referrals into the clinic as well as existing pre-ART patients will be confirmed with an HIV test administered by IDCC staff as recommended by WHO before initiating ART (World Health Organization, July 2015).

**2)** **Same day ART initiation:** Patients who are not on ART will be offered initiation (or re-initiation) of ART on the day of their first clinic visit if blood draws are possible that day. Adherence counseling, screening for opportunistic infections, and blood draw for baseline pre-ART safety labs will occur on the day of ART initiation. Patients who decline ART initiation (or re-initiation) will be given a 1 month follow-up appointment for re-consideration of ART initiation.

**3) Frequency of clinic follow-up visits:** Patients on ART who are stable (e.g., virally suppressed, no opportunistic infections, keeping appointments, etc.) will be offered a reduced frequency of scheduled visits as compared to the current MASA standards. Patients will be followed according to national program standards for the first year. For stable patients on ART for longer than 12 months, visits with a clinician every 6 months will be implemented. IDCC patients already on ART will follow the streamlined schedule according to their time on ART.

**4) Frequency of visits for laboratory testing:** Routine laboratory testing will be offered to patients according to the national guidelines with the exception of CD4 counts which will only be measured at baseline, after 12 months on ART, and at the discretion of the clinician. Also, wherever possible, patients will have their blood drawn at the time of routine clinic visits rather than on separate days to further reduce the frequency of clinic visits and clinic congestion. All laboratory results will be reviewed by the clinic staff within 4 weeks of blood draws, and patients will be contacted by clinic staff for review of any abnormal lab results.

**5) Frequency of dispensing visits:** Patients who are stable after 12 months of ART (e.g., virally suppressed, no opportunistic infections, keeping appointments, etc.) will be dispensed up to 3 months of ARVs if there is adequate stock in the clinic at the time of dispensing rather than being required to return after only 1 month for a dispensing visit. The pharmacy will update a patient’s next refill appointment in PIMS2/IPMS if more than one month is dispensed. Stable patients will have monthly, bi-monthly or quarterly refill appointments, which may coincide with the semi-annual clinician visits.

**6) Retention Intervention*:*** Resources will be available in CP communities to ensure that all PLHIV are retained in care. The retention intervention will focus efforts on those who have missed treatment appointments and/or pharmacy pick-ups. Persons eligible for retention tracing will be identified by the IDCC staff using their electronic data system.

Existing IDCC patients who have missed appointments or pharmacy pick-ups are contacted by MOH clinic staff by phone or home visits and are asked if they agree to have a community counselor contact them to provide retention support activities. Patients who do not consent to retention support intervention activities are reminded about the importance of staying in care/linking back into care, and thanked for their time (***Linkage,*** ***Retention and Adherence Tracing Verbal Consent Script; Retention Tracing Contact Log***).

Patients who consent to intervention activities receive retention support services by clinic staff and community counselors including SMS reminders of appointments, rescheduling of missed appointments, and tracing and supportive counseling through home visits and telephone calls for defaulters (>14 days late for appointment or pharmacy pick-up) and Lost to Follow Up (LTFU; > 90 days late for an appointment). Clinic data systems are monitored to determine if these patients have relinked to the IDCC (***Retention Tracking Log, Participant Tracing Form***).

**7) Adherence Intervention*:*** IDCCs typically use self-reports, pill counts, pharmacy refill data and viral loads to monitor adherence. These adherence monitoring strategies are promoted in all study sites. All PLHIV on ART in CPCs are offered an increased level of adherence counseling and support above and beyond what is routinely offered within the setting of government clinics. Eligible persons who would benefit from increased adherence support are identified by the clinic staff.

Eligible patients are contacted by MOH clinic staff by phone or home visit (***Retention and Adherence Outreach Script*** and are asked if they agree to have a community counselor contact them to provide adherence support activities (***Linkage, Retention and*** ***Adherence Tracing Verbal Consent Script***). Patients who do not consent to adherence support intervention activities are reminded about the importance of taking ART as prescribed, and thanked for their time.

Patients who consent to intervention activities (***Adherence Intervention Verbal Consent/Assent Script***) receive adherence intervention services including an assessment (***Adherence Assessment Tool*),** assignment of case manager, and additional counseling to assess and resolve individual adherence challenges. In addition, the following services may also be delivered depending upon the person’s circumstances and assessment: referral to social worker; SMS reminders; home visits; pill box issuance; depression screening; support with HIV disclosure; alcohol assessment; treatment buddy/mopati; VL repeat; assignment of earlier appointment; and change in ART regimen (for those in whom side effects/medication acceptability might be mitigated through substitution). All adherence interventions will be documented as will the outcome of the efforts, on the ***Adherence Intervention Tracing Log***.

**Patient CARE in Other Study Clinics or Non-Study Clinics**

Finding solutions so that as many BCPP patients as possible can receive expanded ART initiation is important for reaching ART saturation in CP communities, however the study is constrained and cannot easily perform study activities or install study staff at sites which are not CP community clinics. As a solution, the following options will be offered to patients, if during counseling it is determined that they face challenges to participation in study activities at the IDCC in their own community:

1. *BCPP activities at any CP community clinic:*  Initiation and receipt of ART will be allowed at *any* CP community clinic at which a participant would be able to receive services. Note that the study will not be able to provide transport or any additional compensation for these individuals. Follow-up data from PIMS2 or IPMS will be obtained on these individuals for study purposes from the CPC clinic the participant chooses.
2. *ART initiation activities at own community clinic, followed by transfer to any MOH clinic:* Services including ART initiation will be provided at the participant’s own CPC, followed by facilitated transfer to any MOH HIV care and treatment facility (including those not in study communities) for continued ART. Follow-up data on these individuals will be obtained from routine medical information sources such as PIMS2 or the national HIV data warehouse.

**Identifying Patients for UTT**

To find all eligible PLHIV not currently on ART and initiate them on treatment, BCPP priorities will include approaching the Expanded Treatment Cohort (described in BCPP Protocol 3 version 3.0) in early 2016

to offer ART initiation for those not on ART. Another strategy to identify eligible PLHIV not currently on ART will include data mining of the MOH electronic patient systems to seek PLHIV categorized as “pre-ART” to approach them about immediate ART initiation (***Script for Outreach Pre-ART Clinic Patients***). These two mechanisms in combination with expanded HTC activities outlined earlier will yield the scale-up of ART initiation necessary to meet both treatment and viral suppression targets.

**7.0 LABORATORY METHODS**

Detailed laboratory procedures are described in Standard Operating Procedures (SOPs) that are maintained at each laboratory supporting the study. The BCPP Laboratory Manual provides an overview of routine testing that is conducted at Ministry of Health laboratories and more detailed study specific procedures for testing conducted at CDC, Harvard Chan School, BHP laboratories. In addition, all study staff has received a Field Operations Procedures Manual which serves as a procedural guide during actual specimen and data collection.

**7.1 Overview of Laboratory Methods**

An overview of the types of laboratory tests, sample types, and the location of the testing is provided below. The intervention component of the project uses the following laboratories and locations:

1. BHHRL: MOH regional HIV Reference Lab (Gaborone). BHHRL is a hybrid institution with management shared by Harvard and MOH.
2. NHHRL: MOH regional HIV Reference Lab (Francistown)
3. MOH District and primary Labs (Botswana-multi-sites)
4. MOH clinics in CPC communities
5. Field sites: households and community venues conducting surveys and HTC campaigns
6. CDC: International Laboratory Branch at Centers for Disease Control and Prevention (Atlanta) and CDC Laboratories (Gaborone and Francistown)
7. BHP (Botswana Harvard Partnership Laboratory)
8. Harvard T. H. Chan School of Public Health (Boston)

A minimum laboratory enhancement package has been implemented in laboratories supporting study sites. Each study-associated laboratory adheres to site and study specific SOPs for proper processing, labelling, transport, testing and storage of specimens as outlined in the BCPP Laboratory Manual. The laboratory study team provides guidance and technical support to the laboratories to implement and maintain a quality management system which includes continuous quality improvement, adherence to biosafety, enrollment in EQA programs, review and maintenance of standard operating procedures for laboratory testing, timely procurement and forecasting reagent and supply needs, and documentation of training for all laboratory personnel. Adherence to these standards is routinely monitored by the laboratory study team.

**7.2 Training of Laboratory Personnel**

All laboratory personnel participate in study-specific training prior to study initiation. Training includes an overview of the BCPP study design and study-specific laboratory methodologies (with assessment for competency) in addition to Research Ethics and good clinical laboratory practices including QA/QC (quality assurance/quality control) and laboratory record keeping procedures. Study staff responsible for providing HTC complete the required training curriculum recognized by the Ministry of Health. Study staff members who collect blood samples and conduct laboratory analyses receive training in universal precautions, sample collection, and testing of study samples. Additional refresher trainings are scheduled during the study to ensure consistency and address training needs as they arise.

**7.3 Specimen Collection, Transport, and Processing**

Specimens are collected from participants at study field sites and samples transported to designated laboratories for processing, testing, and storage. Testing may also occur at study field sites.

**Sample Types and Sources**

1. *Capillary blood samples:* Finger prick samples are collected in a microtube using a blade lancet by trained staff at study field sites according to BCPP finger prick SOP and used for the HIV rapid tests.
2. *Venous blood samples:* Several types of venous blood draws occur at the IDCC during routine management of patients, and will be done following MOH procedures for phlebotomy, (including appropriate blood volumes and processing). At the IDCC, venous blood samples will be collected for drug resistance testing per national guidelines.

**7.4 Laboratory testing for HTC campaigns (returned to participant and/or participant file with exception of viral load test)**

1. *HIV Diagnostic Testing:* HIV diagnostic testing will be conducted in the study communities and clinics per national HIV testing guidelines using Botswana government–approved serial rapid tests as outlined in the 2016 testing algorithm in the national treatment guidelines. Other testing algorithms may be substituted if in alignment with national guidelines. Verification will be conducted on all newly diagnosed and known HIV-positive persons before initiating ART according to WHO recommendations (World Health Organization, July 2015). All HIV tests will be conducted according to the manufacturer’s guidelines and BCPP SOPs. If results of the initial serial rapid tests are discordant, then per national guidelines two rapid tests will again be performed in parallel. If the second pair of tests is discordant, the participant will be referred to the clinic for re-testing in 2-4 weeks with a referral recommending ELISA testing. A 3 ml venous blood specimen will be taken at the visit and sent to a reference laboratory for further testing using ELISA (Murex HIV 1.2.0 test, Genetic Systems HIV 1.2 plus O, BioRad HIV-1/HIV-2 plus O EIA,) and/or Western blot). The results from the EIA and/or Western Blot will supersede the discordant and/or indeterminate results obtained in the field. The IDCC Site Coordinator reviews the HTC register at the clinic in PIMS2 or IPMS to determine if HTC campaign participants with indeterminate results received confirmatory testing. HTC participants with indeterminate rapid test results, and who do not have a documented retest at the clinic are referred to a community counselor for tracing and follow-up services.

**7.5 Laboratory Testing for IDCC patients**

Clinical laboratory testing is performed in MOH labs in accordance with the National Guidelines. Testing procedures follow approved standard operating procedures for each laboratory where testing is conducted.

a) *Hematology and Clinical Chemistry*

Basic blood work is used to assess participant health status for initiation and monitoring ARV treatment in HIV-infected individuals; however, ART initiation will occur before all lab results are available. The following tests may be conducted per MOH guidelines:

- *Hematology*: Full Blood Count (FBC) to include hemoglobin, hematocrit, red blood cell count (RBC), mean corpuscular volume (MCV), white blood cell count (WBC), differential WBC, absolute neutrophil count (ANC), and platelets.
- *Clinical Chemistry*:
- Liver Function Tests (LFT): Total bilirubin, AST (SGOT), ALT (SGPT), and alkaline phosphatase.
- Glucose (fasting or non-fasting)
- Electrolytes: sodium and chloride
- Renal Function Tests (RFT): Urea and Creatinine*:* An estimated creatinine clearance
- CrCl in cc/minute can be estimated from formulas using patient sex, age, weight, and serum creatinine (in um/L):

Males: 1.22 x [(140 - age in yrs) x wt (kg)]/ [serum Cr]

Females: 1.037 x [(140 – age in yrs) x wt (kg)]/[serum Cr]

b) *Syphilis testing (RPR and VDRL*): As per Botswana guidelines, non-treponemal tests for syphilis (rapid plasma reagin or Venereal Disease Research Laboratory tests) will be performed according to the manufacturer’s instructions.

c) *CD4:* Point of care and flow-based CD4 methods are used dependent upon availability of CD4 instruments. Testing follows manufacturer’s instructions and national guidelines for use.

d) *HIV-1 RNA Viral Load:* HIV-1 plasma RNA viral load is quantified for monitoring of ART-naïve HIV-infected individuals and monitoring of viral suppression in HIV-infected individuals receiving ART (at 3 and 6 months after ART initiation and every 6 month thereafter – according to current national ART guidelines or per Adherence Intervention Procedures).

e) HIV drug resistance testing: Drug resistance testing is offered per national guidelines and under circumstances described in section 6.11.

f) *Pap smear:*  Pap smears are performed and findings managed according to national guidelines.

g) *Pregnancy Testing*: If pregnancy is suspected but not confirmed by LMP (last menstrual period) or exam, a urine pregnancy test is performed for confirmation of pregnancy as per MOH procedure.

**7.6 Quality Assurance**

Standard operating procedures (SOPs) are used for all tests as well as quality assurance/quality control (QA/QC) procedures. Laboratory staff conducting testing participate in a proficiency testing and 5% of all tests (or as indicated by laboratory specific SOP) are subjected to internal quality control processes. The QA/QC plan includes periodic monthly reviews of EQA performance, collaboration with the CDC laboratories, enrollment in appropriate EQA schemes, and inter-laboratory comparisons. External quality assurance and proficiency testing programs includes: CD4 count, HIV-1 RNA load, Hematology (FBC), and clinical chemistry analytes. Laboratories are monitored throughout the study by the study team and external monitors as requested by the study sponsor. QA for HTC conducted in the communities and through RHT in the clinics include weekly QC exercises for counselors in the field, participation in proficiency testing programs, monitoring and site visits from lab personnel, and bi-weekly monitoring of the concordance rates of HIV serial tests conducted in the field. A small sample of HIV-positive tests may be retested in the lab for QA purposes.

**7.7 Biosafety and Waste Management**

As the transmission of HIV and other blood-borne pathogens can occur through contact with contaminated needles, blood, and blood products, appropriate blood and secretion precautions are employed by all personnel in the drawing of blood and shipping and handling of all specimens for this study, as currently recommended by the United States Centers for Disease Control and Prevention. All infectious specimens are transported in accordance with the IATA guidelines using designated courier services.

**7.8 Specimen Storage and Shipment**

The IDCC Site Coordinator coordinates shipment of specimens as required. Selected samples may be transferred to the CDC (Atlanta) or Harvard Chan School (Boston) laboratories for external quality assurance, viral load, and drug resistance/level testing. Specimens are stored until all protocol-related and quality assurance testing have been completed.

**8.0 DATA SYSTEMS AND MANAGEMENT**

The overall system to capture data, record outcomes and analyze results is an integration of multiple data collection sources for all BCPP interventions into a single research data base or data warehouse. Each BCPP activity has underlying systems of data capture and management which serve as the “system of record” containing the source records for all study data captured within the scope of that activity. A variety of data capture methodologies are used across the BCPP activities including online reference data retrieval (e.g., community maps), paperless point of service/survey capture, and paper and electronic chart/registry abstraction. Process, study participant status, and calculated data are also generated by the data systems of the BCPP activities and exchanged between systems and stored in the research database.

Each system must also interface with other activity data systems to exchange data as required to successfully implement the study. This includes maintaining participant registries, referrals, and key eligibility and status data, as well as underlying denominators within study arms and components. In this section, we provide an overview of the data management for BCPP including data capture, data flow, security, confidentiality, access and quality.

**8.1 Data collection, Capture and Flow**

HTC data forms (***Household Enumeration Form, HTC Intake Form***) are collected with the OpenDataKit (ODK) software using handheld tablets running the Android operating system. HTC counselors are trained in the use of these handheld tablets for electronic data entry at the point of HTC, which includes door-to-door home visits and mobile HTC sites.

Community counselors also record their tracing activities for linkage and retention on the ***Participant Tracing Log*** using these handheld mobile devices.

HTC and Linkage to Care forms are synced to the clinic-level study database at least daily. Completed forms are deleted from the tablets following the sync.

Clinic Site Coordinators manage screening, adherence and retention activities using both the hand-held data devices and OpenClinica.

The MOH has an electronic data system known as the Patient Information Management System 2 or PIMS2. PIMS2 is a SQL-based, client-server application which runs on a local clinic network and has been implemented in BCPP community clinics. PIMS2 captures data for a variety of programs, including: facility-based routine HTC, MC, ANC, maternity, and post-natal care. HIV care and treatment data are regularly extracted from PIMS into the clinic-level study database via a secured Virtual LAN (VLAN) connection between the local clinic network on which PIMS2 operates and the BCPP network.

Some larger BCPP community clinics have migrated to a web-based system to record patient-level data which runs on the Botswana Government’s data network. This Integrated Patient Management System or IPMS captures program data that are similar to PIMS and includes an integrated laboratory information system. IPMS data will be regularly extracted from the IPMS central data repository onto the BCPP central data server.

The MOH strongly encourages the use of PIMS or IPMS where available. Therefore, nurse prescribers, physicians, midwives, lay counselors and other clinic staff within the 30 BCPP communities record patient registration and appointments, and enter consultation, laboratory and pharmacy data into PIMS or IPMS. ***Appendix P1*** outlines the selected data elements transferred from MOH systems to the BCPP central data server.

MC staff members responsible for the coordinating linkage of eligible men to MC will distill aggregate data on numbers of total and eligible men transported to MC from the transportation logs and will also engage with service delivery providers to obtain numbers of eligible men served on non-transport days. This data will be entered into an electronic or paper-based custom data collection form monthly.

On a daily basis during the BHS/AHS surveys in all 30 communities, BHS/AHS referral and locator data files are transferred from BHP’s electronic data capture system to the clinic via secure copy on the BCPP network in the clinic and processed into the clinic-level study database. At CPCs, BHS referrals are combined with HTC referrals. In all community clinics, referral data is used to produce operational reports for use in the clinic. In CPCs, an encrypted flash drive is used to retrieve updated referral files from the clinic’s study database and to transfer updated data from the MC client tracking tool to the clinic-level study database.

On a nightly basis, data from all clinic-level study databases is copied to the BCPP central data server in Gaborone over the secure BCPP VPN. As needed, the BCPP senior data manager in Gaborone will work with IDCC Site Coordinators or other clinic level staff to reconcile data issues.

The Research Database is designed in PostgreSQL and housed on the BCPP central data server at the CDC-Botswana Gaborone office. It is populated with data pulled up from all the clinic level study databases as well as data from IPMS, PIMS, CD4 data from PIMAs, and baseline, annual and end of study survey data transferred from BHP’s EDC via secure copy.

The diagram in Figure 1 offers a schematic representation of the data flows from all sources described above to the central BCPP data warehouse for the intervention communities (CPCs). For all PLHIV in the study communities we obtain clinical data from the existing MOH’s electronic patient data systems (PIMS or IPMS).

**Figure 1: Data Flow diagram for CPCs**

**8.2 Data Security**

The CDC Data Manager is responsible the security of BCPP data collected for the Intervention protocol. The Botswana MOH is responsible for the security of data collected through routine MOH systems, including PIMS and IPMS. All BCPP-specific systems supporting clinic data reside within the clinic network where a firewall, encryption keys, user logins, and physical security and access, and routine data back-up measures have been put into place to ensure data security and integrity.

Laptops and tablets used for data capture and processing are encrypted and require user logins to prevent unauthorized users from accessing data. Laptops, tablets and all study related paper-based forms and logs are stored in locked cabinets or locked trunks when not in active use. The clinic laptops which serve as the local clinic-level study databases are locked in secure cabinets. Daily data transfers from tablets and the MC Epi Info system minimize the risk of data loss should the device be lost or corrupted. Clinic-level study databases are backed up on to the local encrypted hard drive and the backup files are transferred to the BCPP central data server in Gaborone using secure copy. The backups from all of the clinic-level study databases and the central data server will be backed up to tape daily. The BCPP central data server at the CDC-Botswana Gaborone office is located in a locked, climate controlled server room managed by CDC IT staff in compliance with CDC IT policies.

A virtual private network (VPN) connects each clinic-level study database to the BCPP data center. The VPN provides a secure network over which data can be transmitted and through which data managers and IT staff can access clinic systems. VPN accounts are provided to study staff as needed and CDC DM lead approval is required for access to study data systems managed by CDC. Within the BCPP network, access to devices, databases, and directories are managed based on user roles. Data in transit over the BCPP network is encrypted, password protected and transmitted through secure connections using secure-shell (SSH) protocols; encrypted, password protected memory sticks are used for data transfer when direct network connections are not available.

The Botswana MOH is responsible for the security of PIMS and IPMS data. PIMS resides on secure local networks at each clinic and IPMS runs on the secure Government Data Network (GDN). Both the local network and the GDN use PIMS are regularly backed up via secure memory sticks. IPMS backups are managed through the IPMS central data store in compliance with Botswana MOH IT policies. CDC DM and IT will work with MOH counterparts to strengthen data security policies and practices.

**8.3 Data quality**

ODK Surveys on the tablets and EpiInfo have built-in validation tools to minimize data entry errors. These include range restrictions for numeric data, pre-defined values (code sets) for categorical data, and logic checks. Valid responses are required for key fields to ensure completeness and skip patterns will be used to prevent extraneous data entry and encourage proper form completion. Error messages and caution notices will be used to alert staff when they enter faulty data so that they are aware and can correct the problem.

HTC and linkage to care staff receive training on both the contents of the tools and use of the tablets and ODK software. Supervisors review the data forms for completeness and supervise corrections prior to synching of the tablets. SMC staff will receive training on proper completion of MOH tools and use of the Epi Info client tracker. Data managers will monitor system performance throughout implementation.

**8.4 Confidentiality of data**

CDC DM is responsible for protecting the privacy of all study participants and ensuring the proper handling of all personal health information and contact information collected for BCPP under Protocol 3. All components of the distributed data systems implement both electronic and physical security procedures for “data in use” within secure networks and devices. Encryption is implemented to render electronically stored subject identity and personal health information unusable, unreadable, or indecipherable to unauthorized individuals for “data in motion” and “data at rest”. Encrypted personally identifiable information (PII) will be transmitted within the BCPP system by way of the secured VPN only.

PII, including name, national identity card number (Omang) and locator data (address, phone numbers), is collected as part of HTC to facilitate referral and linkage of clients with services (e.g. IDCC, ANC, SMC). BHS collects similar information and includes necessary PII in the transfer of referral and locator data to the clinic. PII is collected as a part of patient registration in both PIMS and IPMS as required by MOH to ensure patients are eligible to receive services.

PII maintained in the clinic study databases are not transferred to the central database on the BCPP server; Omang is encrypted with a one-way hash prior to transfer to the BCPP server for the purpose of linking data across data systems. Where there is consent from the client to access medical records or a waiver of consent to extract identified data from the clinic systems (PIMS and IPMS), clinic data can be linked with BHS referrals and HTC data using the hashed Omang.

All study staff are trained on data confidentiality. Site staff access to PII and locator data is limited to health care providing staff which are required to reliably identify a participant, and staff engaged in linking patients consenting for contact with services. A minimal number of staff have access to the study databases where PII is stored and the operational reports which present PII to site staff; DM can access these data when required for system maintenance or to provide user support.

**9.0 DATA AND ANALYSIS PLANS**

The primary objective of the Intervention Protocol is to implement and describe uptake of Combination Prevention interventions in CPCs. Secondary objectives will describe retention in the clinical cascade of care from HIV diagnosis to viral suppression among all HIV-infected persons. The key indicators for analyzing results of BCPP objectives are outlined in Table 2.

.

**Table 2. BCPP Key Indicators**

| Indicator Name | Coverage vs. BCPP Performance | Numerator | Denominator |
| --- | --- | --- | --- |
| 1. % of HIV+ Adults* who Know their Status | Coverage | # of HIV+ Adults estimated to know their HIV status | # of Adults estimated to be HIV+ |
| 1. % of HIV+ Adults in Care | Coverage | # of HIV+ Adults in Care | # of HIV+ Adults estimated to know their HIV status |
| 1. % of HIV+ Adults on ART | Coverage | #of HIV+ Adults on ART | # of HIV+ Adults estimated to know their HIV status |
| 1. % of HIV+ Adults with Viral Suppression | Coverage | # of HIV+ Adults with VL<1000Copies/ML | #of HIV+ Adults on ART |
| 1. % of HIV+ Adults Identified through BCPP who know their status | BCPP Performance | # of HIV+ Adults Identified (new & known) through BCPP who know their status | # of Adults estimated to be HIV+ |
| 1. % of PLHIV Newly Diagnosed through BCPP | BCPP Performance | # of Adults Identified as Newly Diagnosed HIV+ through BCPP | # of HIV+ Adults Identified (new & known) through BCPP who know their status |
| 1. % of PLHIV Linked to Care | BCPP Performance | # of HIV+ Adults Identified as Newly Diagnosed or Not Currently in Care at IDCC who were seen at IDCC within 90 and 180 days of Interview | # HIV+ Adults Identified as Newly Diagnosed or Currently Not in Care at IDCC |
| 1. % of PLHIV Initiating (or re-starting) ART | BCPP Performance | # of Eligible HIV+ Adults Identified Not on ART who Start or Re-Start ART | # of HIV+ Adults Identified not on ART |
| 1. % of PLHIV Retained on ART (12 months Post ART Start or Restart) | BCPP Performance | # of HIV+ Adults Identified Not on ART who Start or Re-Start ART and Remain on ART after 12 Months | # of Eligible HIV+ Adults Identified Not on ART who Start or Re-Start ART |
| 1. % of PLHIV on ART with Viral Suppression (12 Months post ART Initiation or Restart) | BCPP Performance | # of HIV+ Adults Identified Not on ART who Start or Re-Start ART and have VL<1000Copies/ML after 12 Months | # of Eligible HIV+ Adults Identified Not on ART who Start or Re-Start ART |

In the section below, data and the data sources for the outcomes of interest are identified for each intervention within the combination prevention package of services (HTC and Linkage to care, HIV care and treatment, MC and PMTCT services). In addition, the data analysis plans are provided for the study objectives.

**9.1 HIV Testing and Counseling and Linkage to Care**

The HTC services aim to maximize the number of PLHIV who know their status within the community. Predictors of HTC uptake will be examined including demographics, prior testing history, partner status, etc. Participants reached through mobile vs home-based testing will be described including age and gender. Characteristics of HIV-positive persons identified will be described, including but not limited to demographics, testing history, new or previous HIV diagnosis, history of HIV care, CD4 count, and ART status. Data sources will include the ***Household Enumeration Form,*** ***HTC Intake Form, and BHS*** collected during the HTC activities. In addition, facility-based testing will be monitored in CPCs through RHT data in PIMS2 or IPMS.

All HIV-positive participants given appointments at the HIV care and treatment clinic in the study communities are tracked to ensure they link to care. Those who keep appointments at the study clinic within 90 and up to 180 days of the BCPP interview as recorded in PIMS2 or IPMS are considered “linked” to care. Those participants who report they are receiving care at another clinic outside the study community will be verified by PIMS2 or IPMS and will be considered linked to care if that have an IDCC appointment within 90 and up to 180 days of their BCPP interview date. In addition, at one year from the interview data, electronic data systems will be checked to determine if participants who did not link or register within 90 days have registered in care.

Predictors of linkage to care will be examined including but not limited to, demographics, new or previous diagnosis, CD4 count, and time to first appointment from interview. In addition, rates of linkage to care will be compared among participants who received interventions services and those who did not.

Data sources include referrals from HTC and BHS (***HTC Intake***, ***BHS***), PIMS2 and IPMS for details of registration into care and treatment, and the ***Participant Tracing Log*** where all linkage to care follow-up activities are recorded***.*** In addition, the Ministry of Health’s Central Data Warehouse will be queried to identify (or confirm self-reported enrollment) of community residents who register for HIV care and treatment outside of the local study community.

Further, estimations of the operational costs associated with the expanded HTC and linkage to care interventions paired with the total cost by intervention with their corresponding BCPP performance indicator will be used to estimate the average costs per person. The financial costing approach is described below, in the cost analysis plan.

**9.2 Care and Treatment**

For HIV care and treatment objectives, the overall number of PLHIV enrolled in the IDCC and on ART including community residents getting care at clinics outside the community will be described. Specific focus will be given to examination of patterns of health care utilization by residents and non-residents of the community. The key outcomes for analysis are ART initiation, adherence, retention and virologic suppression. We will also describe the laboratory results showing toxicity, and adverse events among the clinic population on ART.  ART initiation will be measured by start date of ART among all HIV-positive patients initiated on ART as recorded in the electronic patient management system.  Length of time from BCPP interview or encounter to date of initiation on ART will be recorded and monitored. ART coverage will be measured by the number of HIV-positive community residents receiving ART out of the estimated number of HIV-positive community residents.

Adherence levels will be assessed through self-reports, pill counts and pharmacy refill data from the electronic data systems and also from the adherence intervention tools. Based on the self-reports, pill counts and pharmacy refill data, an aggregate measure of adherence will be calculated (e.g., good, limited or poor). Adherence levels at a community clinic will be measured by the percentage of clinic patients with an adherence assessment of good, limited and poor adherence. Retention of HIV-positive persons in care and on treatment will be measured during a defined period of time (e.g. 12 and 24 months). The number of HIV-positive clinic patients who are community residents and who remain in care (had a clinic visit within 6 months) and on treatment (pharmacy pick up and clinic visit within 3 months) during a defined period among all patients from the community who were registered at the clinic during that time will be considered retained in care or on treatment. The number of patients who missed clinic visits, defaulted from care and were lost to follow up will also be recorded from the electronic patient management system during a defined period. Virologic suppression will be assessed from the electronic data systems and will be defined as those HIV-positive patients on ART with a viral load of less than or equal to 400 copies/ml among all patients on ART.

Predictors of retention, ART initiation, adherence and virologic suppression will be examined. As the inverse of retention, predictors of loss to follow will be examined.  For those on ART, predictors of defaulting on ART will be examined.

All clinic patients will have data captured in the existing MOH electronic patient management systems. Clinical outcomes, including but not limited to ART eligibility and initiation, WHO stage, lab results, death, adherence, retention, viral suppression, and adverse events, will be assessed using routinely collected clinical data that is captured in the electronic medical record (see Appendix P1). In addition, the Ministry of Health’s Central Data Warehouse or other sources of routine medical information will be queried to identify clinic participation, ART eligibility, ART initiation and virologic suppression among community residents receiving services outside of the local study community clinic. Secondary descriptive analyses for key clinical indicators will be conducted on these data. In the CPCs, retention intervention data will be recorded on the ***Participant Tracing Log,* and** adherence intervention data will be recorded on the ***Adherence Assessment Tool*** and the ***Adherence Intervention Tracing Form***.

**9.3 PMTCT Services**

Rates of 3^rd^ trimester retesting among pregnant women will be assessed using the MOH Antenatal Care and Maternity registers and the MOH patient management systems***.*** Among HIV-negative pregnant women enrolled in ANC care, the percent who were retested for HIV between 32-36 weeks will be recorded and monitored over the course of the study.

Rates of linkage to care among HIV-positive post-partum women will be reported. Once discharged from ANC care, HIV-positive post-partum women will be given appointments at the local IDCC. Those who kept the appointments and registered at the clinic as recorded in the electronic patient management system will be “linked” to care. Those who were previously registered patients in the IDCC who re-link to care will be considered re-linked. Predictors of linkage to care among HIV-positive post-partum women will be examined including but not limited to, demographics, time since diagnosis, CD4 count, viral load, and time since delivery. In addition, rates of linkage to care will be compared among participants who received intervention services and those who did not. Retention in care among post-partum women will be measured the same as among all HIV-positive patients in the IDCCs (see above) and recorded in the electronic patient management system. Rates of retention on ART in post-partum women at one year post-delivery will be described. In addition, predictors of post-partum women who default from ART will be examined including demographics, time since diagnosis, time since delivery, CD4 count, and viral load.

Tracing activities with HIV-positive post-partum women referred to the HIV care and treatment clinic will be recorded on the ***Participant Tracing Log***. The MOH patient management system will be used to verify registration of post-partum women in the IDCC. In addition, the Ministry of Health’s Central Data Warehouse will be queried to identify women who temporarily access care and treatment services outside of the local study community during their period of postpartum confinement.

**9.4 Male Circumcision**

Through linkage to MC services, the MC program aims to maximize coverage of circumcision among males, which is defined as the percentage of HIV-uninfected men (aged 16-49) residing in CPC communities who are circumcised. Community-level MC uptake among all and eligible men will be reported. ***BHS*** and ***ESS*** surveys will be used to obtain cross-sectional true estimates of MC coverage, based on self-report. These surveys are described in the BCPP Evaluation Protocol (Protocol 1). Further, MC coverage will be estimated using records of MCs performed as well as the baseline MC prevalence reported by the HTC and BHS campaigns. MC coverage will be evaluated based on the most recent census estimates of the study community population as well as on the enumerated study population.

**9.5 ANALYSIS PLANS**

For the primary outcomes, uptake of the CP interventions, we will estimate the percentage of participants eligible for and receiving the intervention using BHS and HTC data to identify those eligible, and BHS, HTC and clinic data to identify those receiving interventions. Confidence limits for each outcome will be estimated using robust standard errors to account for within-community clustering.

The first secondary objective is to examine demographic, clinical, and social/behavioral facilitators and barriers to uptake of CP interventions. We will fit Generalized Estimating Equations (GEE) models with robust standard errors to account for the clustering within communities. Independent variables for these models will be chosen based on prior literature and hypotheses about how various demographic, clinical and social/behavioral factors impact uptake of HTC, PMTCT, ART and MC. For MC, this type of analysis will only be possible for year 1 interventions because individual-level data on men obtaining MC will not be collected in subsequent years. Variables will be chosen a priori based on theoretical justification rather than statistical results and all chosen variables retained in the model to adjust for confounding.

To compare clinical outcomes, including viral suppression, among HIV-positive persons in CPCs who initiate expanded ART to those who initiate ART according to national guidelines, we will use tests appropriate to clustered data and the distribution of the outcomes of interest. For example, for viral suppression, we will use GEE models to account for the clustering within communities and test for a difference between groups (those initiating expanded ART vs. those who initiate according to guidelines). Since each community will contain both of these groups of participants, clustering has less impact on statistical tests, but still should be taken into account in the analysis to provide appropriate effect estimates and confidence limits.

To estimate the percentage of participants retained on ART among HIV-positive pregnant or breastfeeding women who initiate Option B+ (indefinite ART post-delivery), we will determine the number of HIV-positive pregnant women who initiate Option B+ during the study using BHS, HTC and clinic data, and determine the number retained on ART using clinic data. Confidence limits will be estimated using robust standard errors as for the primary outcome. Three costing studies of HTC and LTC services are planned. First, estimates of costs of home-based testing and mobile testing during the community-based HTC campaigns, including the cost per person and per new HIV case identified will be compared. Second, incremental costs and outcomes of strengthening facility-based routine testing relative to the standard of care will be estimated. Lastly, to assess the impact of the expanded linkage activities, estimates of the costs and outcomes of these tracking & tracing services will be calculated. Outcomes are defined as the number of new or previously diagnosed HIV-infected persons not in care, who register at an IDCC without engaging tracking and tracing services, and who register at an IDCC within 90 and 180 days of their HTC encounter.

A mixed top-down and micro-costing approach will be used to estimate costs. All relevant direct medical costs such as labor and materials, and direct non-medical costs such as program support and administrative overhead will be considered.

Analyses will be conducted from the perspective of the health care system indicating that the costs included would be those typically associated with the providers and payers of care, and would exclude the costs and productivity losses incurred by patients and their informal caregivers. The most significant cost component is expected to be labor, for which the main categories include: HTC lay counselors and supervisors, LTC Coordinators, drivers, and community mobilizers.

Labor time will be estimated by analyzing data records on clients seen, then confirmed through communication with staff and managers, and, as needed, through observation. Other cost items will be estimated through a combination of resource utilization and expenditure records. Any research related expenses will be removed in order for the resulting cost analyses to be useful for the projection of operational program costs. Costs of tests conducted through the baseline and annual household surveys administered by the Botswana Harvard Partnership will not be considered.

Cost and outcome data will be collected, retrospectively, from routine monitoring program data stored in the central study database and from actual program expenditures incurred over the period corresponding to the HTC and LTC programmatic efforts from all BCPP communities. The analytic horizon will begin in November 2013 and correspond to the period during which the HTC campaign and LTC program are delivered in a community. Unit costs (e.g. Cost per tested, Cost per new diagnosis, Cost per person linked) will estimated by dividing total costs by the relevant BCPP performance indicator. Where applicable any future costs will be discounted to their present value at a standard rate of 3%. As needed, costs will be converted from Botswana Pula to US dollars. Costs will be presented in 2015 US dollars. We will conduct univariate sensitivity analysis to identify the inputs which most impact the results and gauge the robustness of the conclusions. Data analysis will be conducted using Microsoft Excel and SAS.

**10.0 ETHICAL CONSIDERATIONS**

**10.1** **Ethical Review**

The protocol, informed consent/parental permission forms/assent forms, research participant education and recruitment materials, and other requested documents — and any subsequent modifications — will be reviewed and approved by the ethical review bodies responsible for oversight of research conducted at the study sites. The Institutional Review Boards/Ethical Committees (IRBs/ECs) for CDC and the Health Research & Development Committee, Botswana Ministry of Health will review the protocol at least annually.

**10.2 Informed Consent**

**a) Interventions and strengthened programs:**

HTC**:** National guidelines for obtaining verbal consent prior to receiving HTC services, for adults aged 16 or older, will be followed. Linkage-to-care interventions now fall under the Ministry of Health’s 2016 national treatment guidelines which recommend that all HIV-positive persons identified be referred and tracked to the HIV care and treatment clinic. Therefore, all linkage to care follow up tracking will now be considered standard of care and will not require permission. However, participants who do not link to care and receive tracking and tracing follow up services will have the option to discontinue those services at any time they request. Similarly, active retention tracing of participants late for appointments has been implemented in several ART clinics in Botswana with plans to expand the scope of this program. For Retention Tracing, clinic patient contact information (name, phone number, address), OMANG (patient medical ID number) and appointment history are abstracted from MOH register and PIMS2 database for retention tracing of clinic patients who have missed scheduled clinic appointments These patients will be contacted by an MOH staff member and permission will be obtained to send BCPP counselors out for tracing and follow up services.

Adherence Intervention: Clinic patients identified as being non-adherent will be asked to consent for the adherence intervention *(****Linkage, Retention &* *Adherence Intervention Verbal Consent***) which requests permission to administer the Adherence Assessment Form.

The informed consent process will occur in the language most comfortable for participants (usually in Setswana) at 8.0 reading level or below. Permission/assent will be documented by having the participant sign the permission/assent form or make a mark if the participant is illiterate; the latter will be witnessed by a third party. A copy of the informed consent/assent form will be offered to the participant, and if applicable, parent or legal guardian giving permission for a minor to participate. Staff will ensure that the participant takes a copy of a study contact card that contains study information and telephone numbers if the participant does not wish to take a copy of the permission/assent form.

**b) Special Populations**

*Minors:* Minors (participants ages 16-17) are only enrolled with the written permission of a parent/legal guardian and if they provide informed assent.

- Under circumstances where clinical or study staff suspect, or there is a reported incident of sexual abuse of a person under age 18, the study staff member must inform the relevant authorities; this is stated in the permission/assent forms.
- Referrals will be made to youth services if needed.

**c) Use of Routine Programmatic Data:** A waiver of informed consent was previously granted for the following activities:

1. Medical record use in accordance with 45CFR 46.116 (d) to extract a limited, preselected dataset (Appendix P1) from MOH clinical data systems in the clinic population in Enhanced Care and Combination Prevention Communities. This is done by linking BHS and HTC data with clinic data to monitor the care and treatment cascade from HIV testing and referral through clinic enrollment, ART initiation and adherence, and retention in care. Procedures for extracting, matching between sources, and de-identification of this data are described in Section 8. Limitation of data-to-data elements based on consent/waiver-of-consent will be applied during the transfer of data from the clinics into the study database. Although not all data elements are part of the matching algorithm, data are matched using date of birth and Omang number. Once match is assigned, the data retained for analysis is de-identified in accordance with the consent/waiver-of-consent and the stipulations of the study protocols.
2. A waiver of informed consent was previously granted for the following: a) data from the HTC Intake questionnaire that is collected as part of HTC programmatic services in households and tent venues in the Combination Prevention Communities, and b) abstraction of aggregate program data from MC and HTC facilities.

The waiver is appropriate because (1) the analysis involves no more than minimal risk to human subjects, and personal identifiers are encrypted for data handlers and analysts; (2) the evaluation does not adversely affect the rights and welfare of the subjects; (3) the evaluation cannot practicably be carried out without the waiver because it is not possible to obtain consent from all service recipients without infringing on the ability to carry out the program in an efficient manner; and (4) if consent for routine data use from all participants was requested, this might reasonably constitute an unnecessary waste of an individual’s time. The types of consents being requested for activities in this study are described in Table 3.

**Table 3: Summary of approach to consent for various components of the study**

| **Project component** | **Targeted subjects** | **Activity** | **Consent** | **Comment** |
| --- | --- | --- | --- | --- |
| HTC | Persons not documented to be HIV-infected | Routine HIV counseling and testing | Verbal consent |  |
| Programmatic Interventions | Eligible residents of study communities | Use of testing (rapid test) and intake data from HTC, linkage to HIV care, TB screening, retention support and PMTCT services | Waiver of consent/assent granted | 45 CFR 46.116 (d) |
| Use of routine- clinical data | Clinic population, all communities | Use of routinely-collected, program data-see Appendix P1 | Waiver of consent/assent granted | 45 CFR 46.116 (d) |
| Clinic Patient Retention and Adherence | IDCC patient population in CPCs | Data collection and permission to follow-up | Linkage, Retention & Adherence Tracing Verbal Consent Script |  |
|  |  |  |  |  |

**10.3 Risks**

The risks, discomforts, and inconveniences that could be posed to subjects participating in this project include the following:

- **HTC Participants:**
- Blood drawing may cause pain and bruising, and rarely, infection at the site where blood is drawn. Study participants may feel lightheaded or faint as a result.
- Repeated HTC may cause stress, anxiety, or embarrassment. Participants are likely to become distressed if they learn they are HIV positive.
- Participants may become embarrassed, worried, or anxious when discussing their sexual practices, HIV risk factors, ways to protect their partner(s) against HIV, and/or ways to prevent acquiring HIV infection.
- Participants may experience discrimination as a result of being perceived by others to be HIV-infected.
- **Other Strengthened Program Interventions:**
- Other strengthened interventions, including linkage for HIV care, ANC, TB screening, and retention support all carry very minimal risk to participants.
- **Persons starting ART:** In addition to the risks described above for survey participants, risks for patients initiating ART include:

Adults starting ART are at risk of ART-related adverse events (e.g., hepatitis, severe skin rash, renal dysfunction, drug resistance, need for regimen change, etc.).

**10.4 Benefits of Research Participation**

- **HTC:** Potential health benefits include: earlier HTC delivered to their place of residence, earlier diagnosis of HIV-infection with subsequent earlier enrollment in care and treatment or PMTCT, earlier awareness of the importance of annual HTC and risk reduction practices for adults who test HIV-negative, and earlier awareness of the benefits of MC for HIV negative males.
- **Other Strengthened Program Interventions:** Evidence for health benefits of linkage-to-care and adherence/ retention interventions are described in Section 6.
- **UTT:** The benefits of earlier initiation of ART might include improved survival and reduction in incidence of adverse events [such as serious non-AIDS events like myocardial infarction, or AIDS-related morbidity (e.g., a new WHO stage III/IV condition)] as well as decreased likelihood of transmitting HIV to others.

**10.5 Confidentiality**

No information concerning the study or the data will be released to any unauthorized third party, without prior written approval of the participant, except as necessary for monitoring by the IRBs, the study sponsor, OHRP, or the Botswana Ministry of Health. No data with subject identifiers will be released.

- **HTC, linkage to care, and HIV care and treatment following HIV diagnosis:**

Project staff visiting households for study visits will identify an appropriately private space to counsel participants, perform HIV tests, and collect individual data. If private space is not available, or the participant does not wish to carry out study activities in the home, an appointment will be made to visit one of the mobile units or study clinics instead. All study documents, including any papers or files containing personal contact information (such as telephone numbers and addresses), will be stored in a secure location and electronic data will be password protected. Full Drive Encryption will be implemented at the hardware layer of all devices storing protected health information. Only designated study staff will have access to study records.

- **Linkage to MC and MC**

MC mobilization will be conducted according to national standards for mobilization. These standards include protections for transport registers and mobilization registers with PII.

**IDCC Care and Treatment:**

Care at the clinic will be provided in adherence with MOH confidentiality guidelines. Consultations between patients and clinic/research staff will occur in a private location. All consent forms kept by investigators are stored in locked steel cabinets at the clinic or on password-protected electronic devices (e.g., tablets). All paper forms are stored in locked steel cabinets and transported in sealed boxes to Gaborone. All data collected via MOH data systems or entered from paper questionnaires are kept securely on password-protected log-in screens.

**10.6 Protocol Compliance and Compliance with UN Convention on the Rights of the Child**

Study staff will adhere to the ethical principles that are reflected in the Declaration of Helsinki (e.g., ICH E6, 45 CFR 46). The study will be conducted in accordance with the Botswana and Sponsor regulations.

The US government is a signatory on the UN Convention on the Rights of the Child (defined as anyone under the age of 18) which requires that any research study or program activity which includes children that are identified as engaged in sex work, trafficked or victims of violence make special provisions for referral of these individuals to services. BCPP will report suspected cases to designated service providers (local police or social workers) as outlined in the Botswana National Guidelines for reporting child abuse or neglect. Contact information for the local police and social workers, and procedures for reporting suspected cases of child abuse or neglect are described in study specific SOP in the BCPP Study Manual. Local police and social workers will be contacted prior to the initiation of study activities in the community to ensure they are aware of the study activities in the community and the procedures that will be followed should BCPP staff encounter suspected cases of abuse or neglect. As per national guidelines, reports of suspected abuse or neglect will be made to local authorities within 24 hours. Study personnel who may come in contact with these study participants will receive training on the appropriate procedures for handling these cases if they arise during the course of the study and training on the SOP documented in the regulatory files. The study will retain documentation of all reports that includes date of report, age of participant, and the name and type of service provider. No identified information will be retained in the documentation; documentation will be retained by the study for 2 years after study close-out. The informed consents for the study contain language that makes clear to the participant what is required to be reported to authorities and the impact on confidentiality.

**11.0 STUDY SAFETY AND MONITORING PLAN**

11.1 Reporting Adverse Events

Expedited Adverse Event (EAE) Reporting Requirements for this Study:

The study team will report in real-time, to overseeing IRB's and to sponsors, any AE following any exposure to the study interventions that **is BOTH possibly related to study intervention and unexpected, and which is a Serious Adverse Event (SAE).** An SAE is defined as any untoward occurrence that:

- - Results in death
  - Is life-threatening (grade 4)
  - Requires hospitalization or prolongation of hospitalization
  - Results in persistent or significant disability/incapacity
  - Is a congenital anomaly/birth defect

This level of reporting is chosen to avoid real-time EAE reporting of all deaths, hospitalizations, etc. occurring in study communities that are definitely not related to study procedures.

Study interventions are defined as:

1. Antiretroviral therapy implemented as per BCPP protocol
2. HIV testing as part of the HTC campaigns
3. Any other study-related activity in which harm (including social harm) could occur.

With regard to ART intervention, EAE reporting will be restricted to SAEs that are possibly related to initiating ART beyond national guidelines.

Timing of EAE reporting:

EAEs will be reported to CDC and HRDC within 2 business days of the study investigators becoming aware of the event.

Grading Severity of Events:

The most current Division of AIDS Table for Grading the Severity of Adult and Pediatric Adverse Events (DAIDS AE Grading Table) dated November 2014 will be used (or more current version, if applicable), and is available on the RSC website ( [http://rsc.tech-res.com/](http://cp.mcafee.com/d/1jWVIg6zqb2rarWtT4PtPqtPhO-qemm6bECQXCzBYQsIIccndFTd7bBPhPtxBUSCyOMUUYUejdXqp6dbbGJMBrpOH2waEnpVsTgKPOVJV_knQ67-LP3RXFZuVtdBUtPNEVd7abTbnjIyCHtN7BgY-F6lK1FJASMrKrKr01NFrF_oCnzZenMS8BjWEq2F_oCnzZendEzHLK6MCIxI_d44WCy0e40Qe63h02Nwid402-gFavCQNNICI-0W8darq2)).

Non-EAE Adverse Event and Incident Reporting:

The risk of adverse events related to the use of ART has been established and there is no indication that enrollees, with intact immune systems, who are receiving ART, are likely to have a higher risk of adverse events.

SAEs that do not meet the criteria for EAEs (i.e. an SAE that is NOT related to the study intervention) will be submitted quarterly to the IRB. A table listing all unrelated SAEs that occurred in the specified quarter will be submitted to the IRB along with a brief narrative of each event. Per discussions with the CDC IRB, SAEs that are unrelated to the study intervention will NOT be reported using the official CDC forms (.1254/.1254S).

Adverse Event Reporting Period:

The expedited AE reporting period for this study is from the time of initial study contact to the last study contact for a participant.

AE reporting process

Reportable events will be documented by the in-country study team who will then notify the IRBs, MOH, CDC-Atlanta, and Harvard Chan School teams. The incident will be discussed and a written action plan will be devised and implemented within 1 week of the initial report, if needed. Written documentation of all events and event resolution will be retained in the study file. Reporting of EAEs, SAEs and AEs to IRBs and the study sponsor will follow institutional and IRB policies. If applicable, a formal report will be sent to the appropriate IRBs using the CDC’s Incident Report Form 1254.

**11.2 DSMB**

Proposed Schedule of DSMB Reviews and Interim Data Analysis

The BCPP is divided into three interlocking protocols (two active protocols, after Protocol 2 was terminated in 2015), and the investigators expect that DSMB review will require information regarding interim study results and study conduct from all three. The combination prevention package under study includes the following interventions that are outside of current SOC in Botswana: provision of ART for all HIV-infected adults regardless of WHO Stage or CD4 count and streamlined ART delivery. Hence, the reports will provide information needed to evaluate the adverse and favorable effects of such interventions on individually treated patients as well as on the communities in which they reside. The study will require at least 6 reviews, DSMB 1-6, proposed to take place at baseline and at the end of years 1, 2, 3, 4, and 5. DSMB 3 and 4 will evaluate intervention rollout and safety. At DSMB 5, the DSMB can recommend stopping for efficacy or extending the study by 1 year should there be low intervention coverage or a lower than expected incidence rate in the Enhanced Care arm. To demonstrate that the study has reasonable power to answer the study questions given information up to the time of each review, investigators will provide the DSMB with assessments of the adequacy of coverage of proposed interventions; estimated conditional power given available incidence information will be provided at DSMB 4 and all subsequent reviews. While there will be separate reports for each of the protocols, each report will make use of the data available from all protocols. Details of the DSMB review plan for each protocol are provided in that protocol, and the full detailed plan is provided in **Appendix C4**.

**11.3 Protocol Violations**

The study will be conducted in full compliance with the protocol. The research team will ensure that volunteers are informed of and understand the risks of their participation in research. Protocol violations and cases of social harm will be documented using the appropriate study forms and reported to IRBs and sponsors in accordance with the institutional policies. Minor protocol violations that do not pose a threat to the health or welfare of participants will be compiled and reported to the IRBs at the time of annual IRB review. Incident reporting is the responsibility of the Principal Investigators of this study. Emergency departures from protocol that eliminate an apparent immediate hazard to participants and are deemed crucial for the safety and well-being of that participant may be instituted for that participant only. In that case the Investigator will contact the IRBs and sponsor as soon as possible to document the reasons for violation and ensuing events.

**11.4 Monitoring Plan**

A complete monitoring plan has been developed and the site will complete a study site activation checklist prior to study initiation. Internal (sponsor) and external monitors will travel to the study site according to a pre-determined monitoring schedule specified in the monitoring plan to conduct general monitoring. The study site will maintain a monitoring log to document all monitoring visits. Investigators and staff will grant access to the monitors to allow inspection and review study documents (e.g., consent forms, data collection forms, other source documents) and pertinent clinic records for confirmation of the study data. Study monitors will also inspect study facilities and documentation and may observe the performance of study procedures.

**12.0** **ADMINISTRATIVE PROCEDURES AND INFORMATION DISSEMINATION**

**12.1 Study Records and Document Retention**

Complete, accurate, and current study records will be maintained and stored in a secure manner throughout the duration of the study and additionally for five years after study close-out. Study records include administrative documentation— including all reports and correspondence relating to the study — as well as documentation related to each participant screened and/or enrolled in the study, including informed consent forms, locator forms, case report forms, notations of all contacts with the participant, and all other source documents. Necessary documents to be maintained by the site before, during and after the study will be in accordance with GCP regulations and guidance. A complete list of needed documentation will be provided prior to initiation of the study and will appear in the study manual.

**12.2 Information Dissemination and Publication Plan**

Study findings will be disseminated through presentations, reports and publication in peer-reviewed journals and other publications. In-country data and country specific information will be made available to national policy-makers, organizational, and implementing partners. A detailed publication and communication plan will be developed that describes the number and types of reports and manuscripts planned for the study and criteria for authorship. Formal presentations at conferences and scientific publications will follow CDC, Harvard Chan School, and MOH guidelines. A data sharing plan will also be developed that details the requirements and process for requesting access to stored data and a samples for sub-studies and future analysis.

**13.0 REFERENCES**

Ananworanich, J., Gayet-Ageron, A., Le Braz, M., Prasithsirikul, W., Chetchotisakd, P., Kiertiburanakul, S., et al. (2006). CD4-guided scheduled treatment interruptions compared with continuous therapy for patients infected with HIV-1: results of the Staccato randomised trial. Lancet, 368(9534), 459–465. doi:10.1016/S0140-6736(06)69153-8.

Attia, S., M. Egger, M. Muller, M. Zwahlen and N. Low (2009). "Sexual transmission of HIV according to viral load and antiretroviral therapy: systematic review and meta-analysis." AIDS 23(11): 1397-1404.

Auld, A. F., N. Wambua, J. Onyango, B. Marston, G. Namulanda, M. Ackers, T. Oluoch, A. Karisa, A. Hightower, R. W. Shiraishi, A. Nakashima and J. Sitienei (2010). "Piloting the use of personal digital assistants for tuberculosis and human immunodeficiency virus surveillance, Kenya, 2007." Int J Tuberc Lung Dis 14(9): 1140-1146.

Auvert, B., D. Taljaard, E. Lagarde, J. Sobngwi-Tambekou, R. Sitta and A. Puren (2005). "Randomized, controlled intervention trial of male circumcision for reduction of HIV infection risk: the ANRS 1265 Trial." PLoS Med 2(11): e298.

Bailey, R. C., S. Moses, C. B. Parker, K. Agot, I. Maclean, J. N. Krieger, C. F. Williams, R. T. Campbell and J. O. Ndinya-Achola (2007). "Male circumcision for HIV prevention in young men in Kisumu, Kenya: a randomised controlled trial." Lancet 369(9562): 643-656.

BAIS (2008). "The Botswana HIV/AIDS Impact Survey III Results. Available: [http://www.gov.bw/Global/NACA%20Ministry/wana/BAIS%20III_Stats%20Press.pdf."](http://www.gov.bw/Global/NACA%20Ministry/wana/BAIS%20III_Stats%20Press.pdf.%22)

BAIS (2012). Botswana National AIDS Coordinating Agency.  The Fourth Botswana HIV/AIDS Impact Survey IV- 2012. Accessed 17 July 2014 at <http://www.naca.gov.bw/sites/default/files/BAIS%20IV%20Protocol.pdf>

Binik, A., C. Weijer, A. D. McRae, J. M. Grimshaw, R. Boruch, J. C. Brehaut, A. Donner, M. P. Eccles, R. Saginur, M. Taljaard and M. Zwarenstein (2011). "Does clinical equipoise apply to cluster randomized trials in health research?" Trials 12: 118.

Blitzstein, J. and P. Diaconis (2006). "A sequential importance sampling algorithm for generating random graphs with prescribed degrees. Available at: <http://www-stat.stanford.edu/~cgates/PERSI/papers/GraphAlgorithm.pdf.>"

Census (2011). "Project Document: 2011 Population and Housing Census. Available at: <http://unstats.un.org/unsd/censuskb20/Attachment453.aspx.>"

Central Statistics Office (2009). Botswana AIDS Impact Survey III (BAIS III), 2008. C. Kebonyethebe Johane. Gaborone, Botswana.

Ciaranello AL, Perez F, Engelsmann B, Walensky RP, Mushavi A, Rusibamayila A*, et al.* Cost-effectiveness of World Health Organization 2010 guidelines for prevention of mother-to-child HIV transmission in Zimbabwe. *Clin Infect Dis* 2013,56:430-446.

Cohen, M. S., Y. Q. Chen, M. McCauley, T. Gamble, M. C. Hosseinipour, N. Kumarasamy, J. G. Hakim, J. Kumwenda, B. Grinsztejn, J. H. Pilotto, S. V. Godbole, S. Mehendale, S. Chariyalertsak, B. R. Santos, K. H. Mayer, I. F. Hoffman, S. H. Eshleman, E. Piwowar-Manning, L. Wang, J. Makhema, L. A. Mills, G. de Bruyn, I. Sanne, J. Eron, J. Gallant, D. Havlir, S. Swindells, H. Ribaudo, V. Elharrar, D. Burns, T. E. Taha, K. Nielsen-Saines, D. Celentano, M. Essex and T. R. Fleming (2011). "Prevention of HIV-1 infection with early antiretroviral therapy." N Engl J Med 365(6): 493-505.

Corbett, E. L., T. Bandason, T. Duong, E. Dauya, B. Makamure, G. J. Churchyard, B. G. Williams, S. S. Munyati, A. E. Butterworth, P. R. Mason, S. Mungofa and R. J. Hayes (2010). "Comparison of two active case-finding strategies for community-based diagnosis of symptomatic smear-positive tuberculosis and control of infectious tuberculosis in Harare, Zimbabwe (DETECTB): a cluster-randomised trial." Lancet 376(9748): 1244-1253.

Craw, J. A., L. I. Gardner, G. Marks, R. C. Rapp, J. Bosshart, W. A. Duffus, A. Rossman, S. L. Coughlin, D. Gruber, L. A. Safford, J. Overton and K. Schmitt (2008). "Brief strengths-based case management promotes entry into HIV medical care: results of the antiretroviral treatment access study-II." J Acquir Immune Defic Syndr 47(5): 597-606.

Curran, K., E. Njeuhmeli, A. Mirelman, K. Dickson, T. Adamu, P. Cherutich, H. Mahler, B. Fimbo, T. K. Mavuso, J. Albertini, L. Fitzgerald, N. Bock, J. Reed, D. Castor and D. Stanton (2011). "Voluntary medical male circumcision: strategies for meeting the human resource needs of scale-up in southern and eastern Africa." PLoS Med 8(11): e1001129.

Daar, E. S., T. Moudgil, R. D. Meyer and D. D. Ho (1991). "Transient high levels of viremia in patients with primary human immunodeficiency virus type 1 infection." N Engl J Med 324(14): 961-964.

Ding, M., P. Tarwater, M. Rodriguez, R. Chatterjee, D. Ratner, Y. Yamamura, P. Roy, J. Mellors, D. Neogi, Y. Chen and P. Gupta (2009). "Estimation of the predictive role of plasma viral load on CD4 decline in HIV-1 subtype C-infected subjects in India." J Acquir Immune Defic Syndr 50(2): 119-125.

Donnell, D., J. M. Baeten, J. Kiarie, K. K. Thomas, W. Stevens, C. R. Cohen, J. McIntyre, J. R. Lingappa, C. Celum and H. S. V. H. I. V. T. S. T. Partners in Prevention (2010). "Heterosexual HIV-1 transmission after initiation of antiretroviral therapy: a prospective cohort analysis." Lancet 375(9731): 2092-2098.

Eaton, J. W., T. B. Hallett and G. P. Garnett (2011). "Concurrent sexual partnerships and primary HIV infection: a critical interaction." AIDS Behav 15(4): 687-692.

Faal, M., N. Naidoo, D. K. Glencross, W. D. Venter and R. Osih (2011). "Providing immediate CD4 count results at HIV testing improves ART initiation." J Acquir Immune Defic Syndr 58(3): e54-59.

Fauci, A. S. and G. K. Folkers (2012). "Toward an AIDS-free generation." JAMA 308(4): 343-344.

Fasawe O, Avila C, Shaffer N, Schouten E, Chimbwandira F, Hoos D*, et al.* Cost-Effectiveness Analysis of Option B+ for HIV Prevention and Treatment of Mothers and Children in Malawi. *PLoS One* 2013,8:e57778.

Fox, M. P. and S. Rosen (2010). "Patient retention in antiretroviral therapy programs up to three years on treatment in sub-Saharan Africa, 2007-2009: systematic review." Trop Med Int Health 15 Suppl 1: 1-15.

Gardner, E. M., M. P. McLees, J. F. Steiner, C. Del Rio and W. J. Burman (2011). "The spectrum of engagement in HIV care and its relevance to test-and-treat strategies for prevention of HIV infection." Clin Infect Dis 52(6): 793-800.

Gardner, L. I., G. Marks, J. Craw, L. Metsch, S. Strathdee, P. Anderson-Mahoney, C. del Rio and G. Antiretroviral Treatment Access Study (2009). "Demographic, psychological, and behavioral modifiers of the Antiretroviral Treatment Access Study (ARTAS) intervention." AIDS Patient Care STDS 23(9): 735-742.

Gardner, L. I., G. Marks, L. R. Metsch, A. M. Loughlin, C. O'Daniels, C. del Rio, P. Anderson-Mahoney, J. D. Wilkinson and A. S. Group (2007). "Psychological and behavioral correlates of entering care for HIV infection: the Antiretroviral Treatment Access Study (ARTAS)." AIDS Patient Care STDS 21(6): 418-425.

Gardner, L. I., L. R. Metsch, P. Anderson-Mahoney, A. M. Loughlin, C. del Rio, S. Strathdee, S. L. Sansom, H. A. Siegal, A. E. Greenberg, S. D. Holmberg, T. Antiretroviral and G. Access Study Study (2005). "Efficacy of a brief case management intervention to link recently diagnosed HIV-infected persons to care." AIDS 19(4): 423-431.

Geng, E. H., D. Nash, A. Kambugu, Y. Zhang, P. Braitstein, K. A. Christopoulos, W. Muyindike, M. B. Bwana, C. T. Yiannoutsos, M. L. Petersen and J. N. Martin (2010). "Retention in care among HIV-infected patients in resource-limited settings: emerging insights and new directions." Curr HIV/AIDS Rep 7(4): 234-244.

Granich, R. M., C. F. Gilks, C. Dye, K. M. De Cock and B. G. Williams (2009). "Universal voluntary HIV testing with immediate antiretroviral therapy as a strategy for elimination of HIV transmission: a mathematical model." Lancet 373(9657): 48-57.

Gray, R. H., G. Kigozi, D. Serwadda, F. Makumbi, S. Watya, F. Nalugoda, N. Kiwanuka, L. H. Moulton, M. A. Chaudhary, M. Z. Chen, N. K. Sewankambo, F. Wabwire-Mangen, M. C. Bacon, C. F. Williams, P. Opendi, S. J. Reynolds, O. Laeyendecker, T. C. Quinn and M. J. Wawer (2007). "Male circumcision for HIV prevention in men in Rakai, Uganda: a randomised trial." Lancet 369(9562): 657-666.

Habicht, J. P., C. G. Victora and J. P. Vaughan (1999). "Evaluation designs for adequacy, plausibility and probability of public health programme performance and impact." Int J Epidemiol 28(1): 10-18.

Halperin, D. T. (2009). "Combination HIV prevention must be based on evidence." Lancet 373(9663): 544-545.

Handcock, M. S., D. R. Hunter, C. T. Butts, S. M. Goodreau and M. Morris (2003). "Software tools for the Statistical Modeling of Network Data. available at: <http://statnet.org.>"

Hayes, R., F. Mosha, A. Nicoll, H. Grosskurth, J. Newell, J. Todd, J. Killewo, J. Rugemalila and D. Mabey (1995). "A community trial of the impact of improved sexually transmitted disease treatment on the HIV epidemic in rural Tanzania: 1. Design." AIDS 9(8): 919-926.

Hayes, R. and L. H. Moulton (2009). Cluster Randomized Trials. Boca Raton, CRC Press.

Helleringer, S. and H. P. Kohler (2007). "Sexual network structure and the spread of HIV in Africa: evidence from Likoma Island, Malawi." AIDS 21(17): 2323-2332.

HHS (2011). "Guidelines for the Use of Antiretroviral Agents in HIV-1-Infected Adults and Adolescents. Avaialble at: <http://www.aidsinfo.nih.gov/ContentFiles/AdultandAdolescentGL.pdf.>"

HHS (2012). "Guidelines for the use of antiretroviral agents in HIV-1-infected adults and adolescents. Available at: <http://infosida.nih.gov/contentfiles/lvguidelines/adultandadolescentglsp.pdf> . Accessed 19 August 2012.".

Hussey, M. A. and J. P. Hughes (2007). "Design and analysis of stepped wedge cluster randomized trials." Contemp Clin Trials 28(2): 182-191.

Jani, I. V., N. E. Sitoe, P. L. Chongo, E. R. Alfai, J. I. Quevedo, O. Tobaiwa, J. D. Lehe and T. F. Peter (2011). "Accurate CD4 T-cell enumeration and antiretroviral drug toxicity monitoring in primary healthcare clinics using point-of-care testing." AIDS 25(6): 807-812.

Jones, J. H. and M. S. Handcock (2003). "An assessment of preferential attachment as a mechanism for human sexual network formation." Proc Biol Sci 270(1520): 1123-1128.

Kaufmann, G. R., P. Cunningham, A. D. Kelleher, J. Zaunders, A. Carr, J. Vizzard, M. Law and D. A. Cooper (1998). "Patterns of viral dynamics during primary human immunodeficiency virus type 1 infection. The Sydney Primary HIV Infection Study Group." J Infect Dis 178(6): 1812-1815.

Kim, A. A., T. Hallett, J. Stover, E. Gouws, J. Musinguzi, P. K. Mureithi, R. Bunnell, J. Hargrove, J. Mermin, R. K. Kaiser, A. Barsigo and P. D. Ghys (2011). "Estimating HIV Incidence among Adults in Kenya and Uganda: A Systematic Comparison of Multiple Methods." PLoS One 6(3): e17535.

Kitahata, M. M., S. J. Gange, A. G. Abraham, B. Merriman, M. S. Saag, A. C. Justice, R. S. Hogg, S. G. Deeks, J. J. Eron, J. T. Brooks, S. B. Rourke, M. J. Gill, R. J. Bosch, J. N. Martin, M. B. Klein, L. P. Jacobson, B. Rodriguez, T. R. Sterling, G. D. Kirk, S. Napravnik, A. R. Rachlis, L. M. Calzavara, M. A. Horberg, M. J. Silverberg, K. A. Gebo, J. J. Goedert, C. A. Benson, A. C. Collier, S. E. Van Rompaey, H. M. Crane, R. G. McKaig, B. Lau, A. M. Freeman, R. D. Moore and N.-A. Investigators (2009). "Effect of early versus deferred antiretroviral therapy for HIV on survival." N Engl J Med 360(18): 1815-1826.

Lawn, S. D., A. D. Harries, B. G. Williams, R. E. Chaisson, E. Losina, K. M. De Cock and R. Wood (2011). "Antiretroviral therapy and the control of HIV-associated tuberculosis. Will ART do it?" Int J Tuberc Lung Dis 15(5): 571-581.

Lester, R. T., P. Ritvo, E. J. Mills, A. Kariri, S. Karanja, M. H. Chung, W. Jack, J. Habyarimana, M. Sadatsafavi, M. Najafzadeh, C. A. Marra, B. Estambale, E. Ngugi, T. B. Ball, L. Thabane, L. J. Gelmon, J. Kimani, M. Ackers and F. A. Plummer (2010). "Effects of a mobile phone short message service on antiretroviral treatment adherence in Kenya (WelTel Kenya1): a randomised trial." Lancet 376(9755): 1838-1845.

Lingappa, J. R., J. P. Hughes, R. S. Wang, J. M. Baeten, C. Celum, G. E. Gray, W. S. Stevens, D. Donnell, M. S. Campbell, C. Farquhar, M. Essex, J. I. Mullins, R. W. Coombs, H. Rees, L. Corey, A. Wald and H. S. V. H. I. V. T. S. T. Partners in Prevention (2010). "Estimating the impact of plasma HIV-1 RNA reductions on heterosexual HIV-1 transmission risk." PLoS One 5(9): e12598.

Little, S. J., A. R. McLean, C. A. Spina, D. D. Richman and D. V. Havlir (1999). "Viral dynamics of acute HIV-1 infection." J Exp Med 190(6): 841-850.

Lyles, R. H., C. Chu, J. W. Mellors, J. B. Margolick, R. Detels, J. V. Giorgi, Q. Al-Shboul and J. P. Phair (1999). "Prognostic value of plasma HIV RNA in the natural history of Pneumocystis carinii pneumonia, cytomegalovirus and Mycobacterium avium complex. Multicenter AIDS Cohort Study." AIDS 13(3): 341-349

.

Mahy, M., J. Stover, K. Kiragu, C. Hayashi, P. Akwara, C. Luo, K. Stanecki, R. Ekpini and N. Shaffer (2010). "What will it take to achieve virtual elimination of mother-to-child transmission of HIV? An assessment of current progress and future needs." Sex Transm Infect 86 Suppl 2: ii48-55.

Mellors, J. W., J. B. Margolick, J. P. Phair, C. R. Rinaldo, R. Detels, L. P. Jacobson and A. Munoz (2007). "Prognostic value of HIV-1 RNA, CD4 cell count, and CD4 Cell count slope for progression to AIDS and death in untreated HIV-1 infection." JAMA 297(21): 2349-2350.

Mellors, J. W., A. Munoz, J. V. Giorgi, J. B. Margolick, C. J. Tassoni, P. Gupta, L. A. Kingsley, J. A. Todd, A. J. Saah, R. Detels, J. P. Phair and C. R. Rinaldo, Jr. (1997). "Plasma viral load and CD4+ lymphocytes as prognostic markers of HIV-1 infection." Ann Intern Med 126(12): 946-954.

Mellors, J. W., C. R. Rinaldo, Jr., P. Gupta, R. M. White, J. A. Todd and L. A. Kingsley (1996). "Prognosis in HIV-1 infection predicted by the quantity of virus in plasma." Science 272(5265): 1167-1170.

Merson, M., N. Padian, T. J. Coates, G. R. Gupta, S. M. Bertozzi, P. Piot, P. Mane, M. Bartos and H. I. V. P. S. A. Lancet (2008). "Combination HIV prevention." Lancet 372(9652): 1805-1806.

Microsoft (2011). "HIV/AIDS Information System Taps Existing Cell Phone Texting Capabilities. Available at: <http://research.microsoft.com/en-us/collaboration/papers/botswana.pdf.>"

MMWR. Impact of an Innovative Approach to Prevent Mother-to-Child Transmission of HIV — Malawi, July 2011–September 2012. March 1, 2013 / 62(08);148-151.

Modjarrad, K., E. Chamot and S. H. Vermund (2008). "Impact of small reductions in plasma HIV RNA levels on the risk of heterosexual transmission and disease progression." AIDS 22(16): 2179-2185.

Montaner, J. S., V. D. Lima, R. Barrios, B. Yip, E. Wood, T. Kerr, K. Shannon, P. R. Harrigan, R. S. Hogg, P. Daly and P. Kendall (2010). "Association of highly active antiretroviral therapy coverage, population viral load, and yearly new HIV diagnoses in British Columbia, Canada: a population-based study." Lancet 376(9740): 532-539.

Mtapuri-Zinyowera, S., M. Chideme, D. Mangwanya, O. Mugurungi, S. Gudukeya, K. Hatzold, A. Mangwiro, G. Bhattacharya, J. Lehe and T. Peter (2010). "Evaluation of the PIMA point-of-care CD4 analyzer in VCT clinics in Zimbabwe." J Acquir Immune Defic Syndr 55(1): 1-7.

Novitsky, V., T. Ndung'u, R. Wang, H. Bussmann, F. Chonco, J. Makhema, V. De Gruttola, B. D. Walker and M. Essex (2011). "Extended high viremics: a substantial fraction of individuals maintain high plasma viral RNA levels after acute HIV-1 subtype C infection." AIDS 25(12): 1515-1522.

Novitsky, V., R. Wang, H. Bussmann, S. Lockman, M. Baum, R. Shapiro, I. Thior, C. Wester, C. W. Wester, A. Ogwu, A. Asmelash, R. Musonda, A. Campa, S. Moyo, E. van Widenfelt, M. Mine, C. Moffat, M. Mmalane, J. Makhema, R. Marlink, P. Gilbert, G. R. Seage, 3rd, V. DeGruttola and M. Essex (2010). "HIV-1 subtype C-infected individuals maintaining high viral load as potential targets for the "test-and-treat" approach to reduce HIV transmission." PLoS One 5(4): e10148.

Padian, N. S., S. I. McCoy, S. Manian, D. Wilson, B. Schwartlander and S. M. Bertozzi (2011). "Evaluation of large-scale combination HIV prevention programs: essential issues." J Acquir Immune Defic Syndr 58(2): e23-28.

PEPFAR (2009). "Celebrating Life: The U.S. President’s Emergency Plan for AIDS Relief 2009 Annual Report to Congress – Highlights. Accessed 3 September 2011 at: <http://www.pepfar.gov/documents/organization/113878.pdf.>"

Piot, P., M. Bartos, H. Larson, D. Zewdie and P. Mane (2008). "Coming to terms with complexity: a call to action for HIV prevention." Lancet 372(9641): 845-859.

Rosen, S. and M. P. Fox (2011). "Retention in HIV Care between Testing and Treatment in Sub-Saharan Africa: A Systematic Review." PLoS Med 8(7): e1001056.

Severe, P., M. A. Juste, A. Ambroise, L. Eliacin, C. Marchand, S. Apollon, A. Edwards, H. Bang, J. Nicotera, C. Godfrey, R. M. Gulick, W. D. Johnson, Jr., J. W. Pape and D. W. Fitzgerald (2010). "Early versus standard antiretroviral therapy for HIV-infected adults in Haiti." N Engl J Med 363(3): 257-265.

Shapiro RL, Kitch D, Ogwu A, Hughes M, Lockman S, Powis K, Souda S, Moffat C, Moyo S, McIntosh K, Widenfelt E, Zwerski S, Mazhani L, Makhema J, Essex M. HIV transmission and 24-month survival in a randomized trial of HAART to prevent MTCT during pregnancy and breastfeeding in Botswana (The Mma Bana Study). AIDS. 2013 (in press).

Stover, J., L. Bollinger and C. Avila (2011). "Estimating the Impact and Cost of the WHO 2010 Recommendations for Antiretroviral Therapy." AIDS Res Treat 2011: 738271

Stover, J., B. Fidzani, B. C. Molomo, T. Moeti and G. Musuka (2008). "Estimated HIV trends and program effects in Botswana." PLoS One 3(11): e3729.

Stover, J., E. L. Korenromp, M. Blakley, R. Komatsu, K. Viisainen, L. Bollinger and R. Atun (2011). "Long-term costs and health impact of continued global fund support for antiretroviral therapy." PLoS One 6(6): e21048.

Tanser, F., T. Bärnighausen, E. Grapsa and M.-L. Newell (2012). "Effect of ART Coverage on Rate of New HIV Infections in a Hyper-endemic, Rural Population: South Africa." 19th Conference on Retroviruses and Opportunistic Infections; March 5-8; Seattle, USA. Available: <http://www.retroconference.org/2012b/Abstracts/45379.htm>. Accessed 4 July 2012.

Tanser, F., T. Bärnighausen, E. Grapsa, J. Zaidi and M. L. Newell (2013). "High Coverage of ART Associated with Decline in Risk of HIV Acquisition in Rural KwaZulu-Natal, South Africa." Science 339(966).

UNAIDS (2010). "Global Report: UNAIDS Report on the Global AIDS Epidemic. Available at: <http://www.unaids.org/globalreport/global_report.htm>. Accessed February 2012.".

Van Rooyen, H., R. V. Barnabas, Z. Phakathi, P. Joseph, M. Krows, T. Hong, J. Baeten and C. Celum (2012). "HIV testing uptake and linkages to HIV treatment through home-based HIV counseling and testing and facilitated referral in Kwa-Zulu Natal, South Africa." CROI 2012.

Walensky, R. P., L. L. Wolf, R. Wood, M. O. Fofana, K. A. Freedberg, N. A. Martinson, A. D. Paltiel, X. Anglaret, M. C. Weinstein and E. Losina (2009). "When to start antiretroviral therapy in resource-limited settings." Ann Intern Med 151(3): 157-166.

WHO (2003). "World health Organization. WHO Guide to Cost-Effectiveness Analysis - 2003." Geneva, Switzerland: WHO; 2003. Accessed August 11, 2010: <http://www.who.int/choice/publications/p_2003_generalised_cea.pdf>.

WHO (2010). World Health Organization. Towards universal access: Scaling up priority HIV/AIDS interventions in the health sector - Progress report 2010. Geneva, Switzerland: WHO; 2010. . Avaliable: <http://wwwwhoint/hiv/pub/2010progressreport/full_report_enpdf>. Accessed 29 September 2010.

WHO (2011). "Global HIV./AIDS Response. Epidemic update and health sector progress towards Universal Access. Available: <http://www.unaids.org/en/media/unaids/contentassets/documents/unaidspublication/2011/20111130_UA_Report_en.pdf>. Accessed 24 August 2012."

WHO (2013) "Consolidated Guidelines on the Use of Antiretroviral Drugs for Treating and Preventing HIV infection" Available: http://apps.who.int/iris/bitstream/10665/85321/1/9789241505727_eng.pdf Accessed 5 December 2013

World Health Organization. Use of Antiretroviral Drugs for Treating Pregnant Women and Preventing HIV Infection in Infants. Programmatic Update. April, 2012. <http://www.who.int/hiv/pub/mtct/programmatic_update2012/en/>.

World health Organization. Consolidated Guidelines on HIV Testing Services. July, 2015. <http://www.who.int/hiv/pub/guidelines/hiv-testing-services/en/>

World Health Organization. Guideline on When to Start Antiretrovoral Therapy and on Pre-Exposure Prophylaxis for HIV. September, 2015.

Writing Committee for the, C. C. (2011). "Timing of HAART initiation and clinical outcomes in human immunodeficiency virus type 1 seroconverters." Arch Intern Med 171(17): 1560-1569.
